# Hydrogen-Bond-Assisted Chalcogen Transfer between Phosphine Selenides and Arsine Oxides

**DOI:** 10.1021/acs.inorgchem.4c05433

**Published:** 2025-05-05

**Authors:** Danil V. Krutin, Semyon V. Tsybulin, Valeriya V. Mulloyarova, Elena Yu. Tupikina, Peter M. Tolstoy, Alexander S. Antonov

**Affiliations:** † Institute of Chemistry, St. Petersburg State University, Universitetskii Pr. 26, 198504 St. Petersburg, Russian Federation; ‡ Institute of Organic Chemistry, University of Regensburg, D-93053 Regensburg, Germany

## Abstract

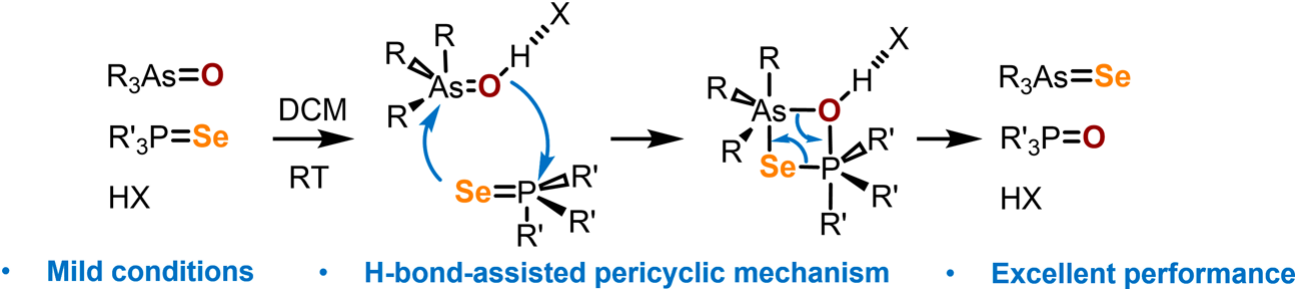

The Brønsted acid catalysis is widely regarded as one of the most effective methods for activating inert substrates and enabling unique reactivity. In this work, we introduce the first example of H-bond-assisted chalcogen exchange between arsine oxides and phosphine selenides under mild conditions, providing a powerful approach to the synthesis of arsine selenides. The reaction proceeds successfully in both protic and aprotic solvents and is accelerated by the presence of any nonaqueous acid. This newly discovered reaction is tested for various arsine oxides R_3_AsO (R = Ph, Et, *n*Bu, *i*Pr) and phosphine selenides R_3_PSe (R = Ph, Me, Et, *t*Bu) and overall demonstrates high conversion, although the use of reagents with bulky substituents significantly hinders its efficiency. The reaction mechanism involves the formation of a four-membered cyclic transition state, which requires overcoming steric and electrostatic repulsion, as well as significant distortion of the reagents’ tetrahedral geometry. Hydrogen bonding with the As=O fragment helps to reduce electrostatic repulsion between the P=Se and As=O groups, making the formation of the cyclic intermediate more favorable.

## Abstract 2

The Brønsted acid catalysis is widely regarded as one of the most effective methods for activating inert substrates and enabling unique reactivity.

## Introduction

In the booming field of Brønsted acid and Brønsted base organocatalysis,^1−5^ there is often little knowledge of a given reaction mechanism. Nuclear magnetic resonance spectroscopy proved itself indispensable for the determination of the structure of H-bonded species,^6,7^ despite complications associated with signal averaging due to the short lifetimes of catalyst–substrate complexes. A correct interpretation of observed NMR parameters in terms of geometry and energy of the hydrogen bonds often requires preliminary investigations of a series of model complexes, in order to construct correlations, such as, for example, the ones previously observed between H-bond energies and bridging proton chemical shifts^8^ or between H-bond geometries and bridging proton chemical shifts^9,10^ or ^31^P NMR chemical shifts of phosphine oxides.^11,12^ Experimental observation of nonaveraged spectral parameters often requires low-temperature measurements designed to slow down chemical exchange processes.^13−15^ This inspires the creation of new molecular probes containing NMR-sensitive nuclei and that are capable of forming complexes with various proton donors.

**Figure 1:**
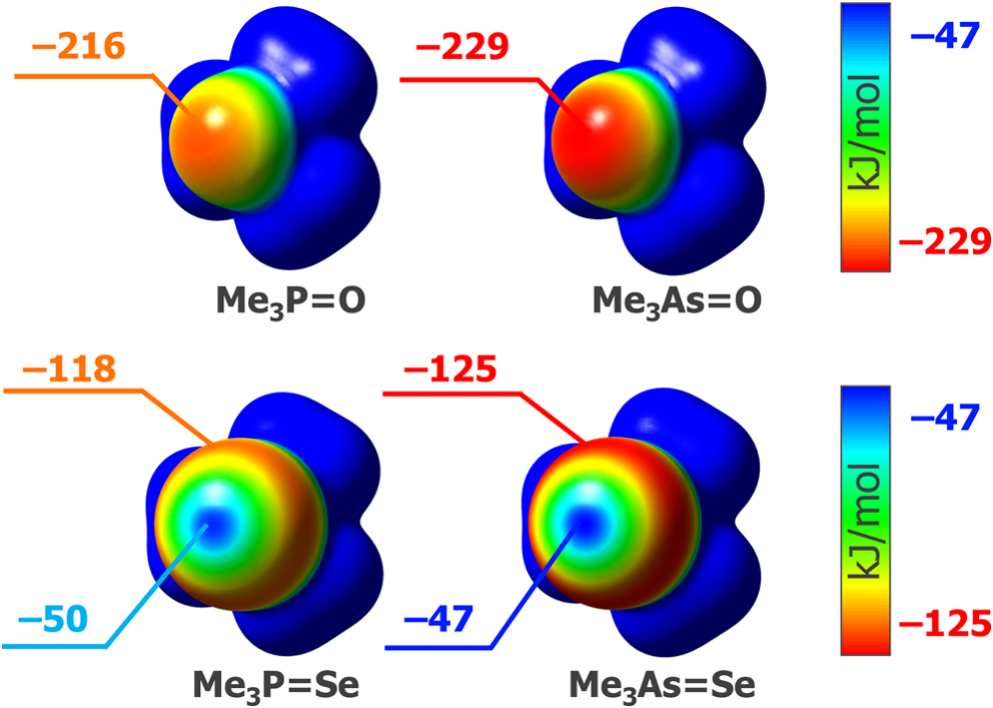
Isosurfaces of electron density (isovalue 0.001 au, van der Waals surface) mapped by electrostatic potential for Me_3_PO, Me_3_AsO, Me_3_PSe, and Me_3_AsSe (PW6B95-GD3/def2-TZVPD).

For the studies of A–H···Ch (Ch–chalcogen) hydrogen bonds, phosphine oxides have proven themselves as a valuable tool due to their strong proton-accepting ability and the presence of ^31^P nuclei (spin 1/2, natural abundance 100%) in the proximity to the proton-accepting site.^11,12,16,17^ Despite the attractive properties of this nucleus, its NMR parameters are often quite sensitive toward the changes in the media, which in many cases prevents a precise NMR-based analysis of hydrogen bonding.^18−20^ It is very tempting to have an NMR-sensitive chalcogen atom directly participating in the hydrogen bond and serving as a spectroscopic probe for changes in the hydrogen bond geometry and energy. One of the best possible candidates for this role is ^77^Se (spin 1/2, natural abundance 7.63%).^21^ Unfortunately, the transition from phosphine oxides (previously established as probes for non-covalent interactions)^12,22−24^ to phosphine selenides dramatically decreases the proton-accepting ability (Figure 1, left), thus making this tool less effective for investigations of hydrogen bonds with weaker proton donors.^25,26^

The only exception is diselenophosphoric acids, which demonstrate extraordinarily strong proton binding by the P=Se moiety.^27^ In contrast, the transition from the P=O to the As=O moiety leads to a decrease of the ESP minima on the van der Waals surface (by 13 kJ/mol; Figure 1, top) and thus to a significant increase of the proton-accepting ability.^28,29^ Based on the calculations, the transition from P=Se to As=Se shows a similar trend: in the region of lone electron pairs of the selenium atom perpendicular to the bond, the value of the ESP decreases by 7 kJ/mol, which means an increase in nucleophilicity or proton-accepting ability. With this in mind, the utilization of arsine selenides as NMR sensors should provide a reasonable balance between the strength of hydrogen bonding and the effectiveness of probing. The first step in this direction is the development of safe and effective methods for the synthesis of arsine selenides.

**Scheme 1:**
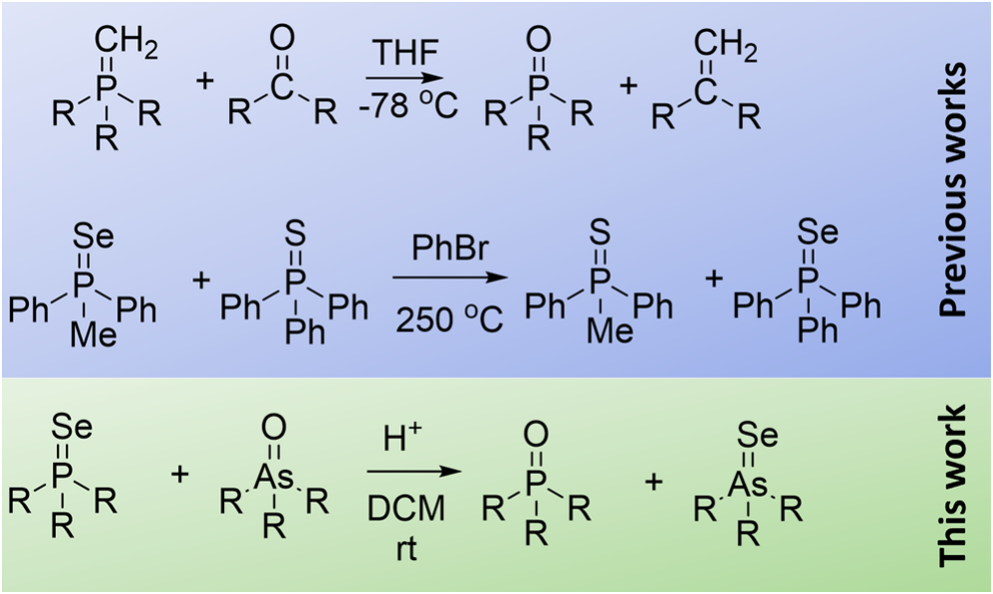
Examples of Chalcogen Exchange in Triorganylphosphine Chalcogenides

The synthesis of arsine selenides is commonly performed by the treatment of arsines with elemental selenium.^30^ Despite its simplicity, this approach requires the utilization of toxic, volatile, and oxygen-sensitive arsines. In contrast, arsine oxides are nonvolatile, stable in air solids, which makes them perfect candidates for substrates for the synthesis of arsine selenides via chalcogen exchange with, for example, phosphine selenides. On the one hand, the strong oxygenophilicity of phosphorus is a driving force of the Wittig reaction, allowing the effective synthesis of alkenes via the exchange of an oxygen atom between the C=O and P=CH_2_ moieties (Scheme 1).^31,32,1^

On the other hand, the chalcogen transfer between phosphine sulfides and phosphine selenides is known.^33^ The harsh conditions required for this exchange significantly limit synthetic application. Keeping the above-mentioned strong proton-accepting ability of arsine oxides in mind, one can expect the possibility to activate the chalcogen exchange between easily available phosphine selenides and arsine oxides via hydrogen bonding. Surprisingly, no attempts to implement this tool have been made to date. Herein, we present the first example of hydrogen-bond-assisted chalcogen exchange between arsine oxides and phosphine selenides under mild conditions as a potent tool for the synthesis of arsine selenides. We demonstrate the crucial role of hydrogen bonding in the promotion of this reactivity and unravel the mechanism of this transformation by means of NMR spectroscopy and quantum chemical calculations.

## Results and Discussion

**Scheme 2:**
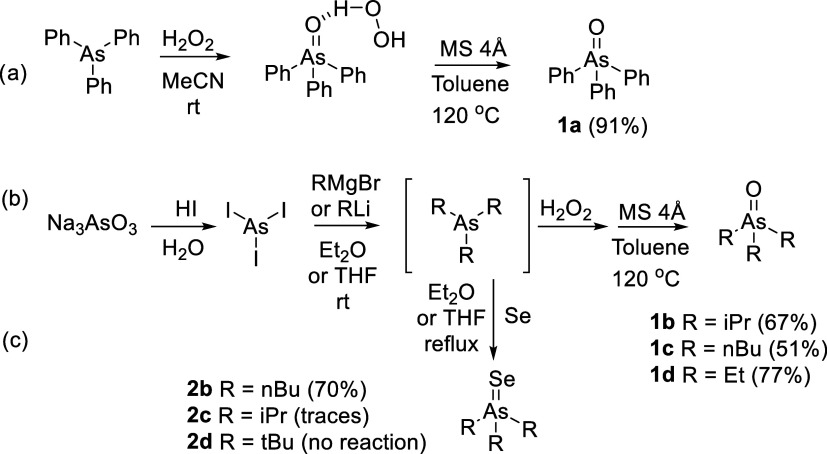
Synthesis of Arsine Oxides (a, b) and Arsine Selenides (c) from Arsines

We started with the synthesis of arsine oxides. Triphenylarsane oxide **1a** was prepared by the oxidation of commercially available triphenylarsane with hydrogen peroxide (Scheme 2a). Due to the volatility and toxicity of trialkylarsines, we developed the *one-pot* synthesis of trialkylarsine oxides **1b**–**d** based on the treatment of AsI_3_ with organomagnesium (or organolithium) reagents, followed by oxidation with hydrogen peroxide (Scheme 2b). AsI_3_ was prepared by the treatment of readily available and cheap sodium *ortho*-arsenite with an aqueous solution of hydrogen iodide. Despite the lesser reactivity of AsI_3_ in comparison with that of AsCl_3_, it is much safer to work with, since it is a nonvolatile solid. In all cases, arsine oxides were obtained in the form of H-bonded complexes with hydrogen peroxide.^34^ In order to prepare free arsine oxides, these complexes were heated with molecular sieves in toluene, as it was previously suggested for complexes of phosphine oxides and arsine oxides with hydrogen peroxide.^35−37^ For the precise investigation of the transformation of arsine oxides into arsine selenides using NMR spectroscopy, we also attempted to use a similar *one-pot* synthesis for arsine selenides **2** based on the oxidation of *in situ* formed arsines with elemental selenium (Scheme 2c). However, although this method was very effective for the preparation of *n*Bu_3_AsSe, its application was unsuccessful for sterically hindered *i*Pr_3_AsSe and *t*Bu_3_AsSe.

**Figure 2:**
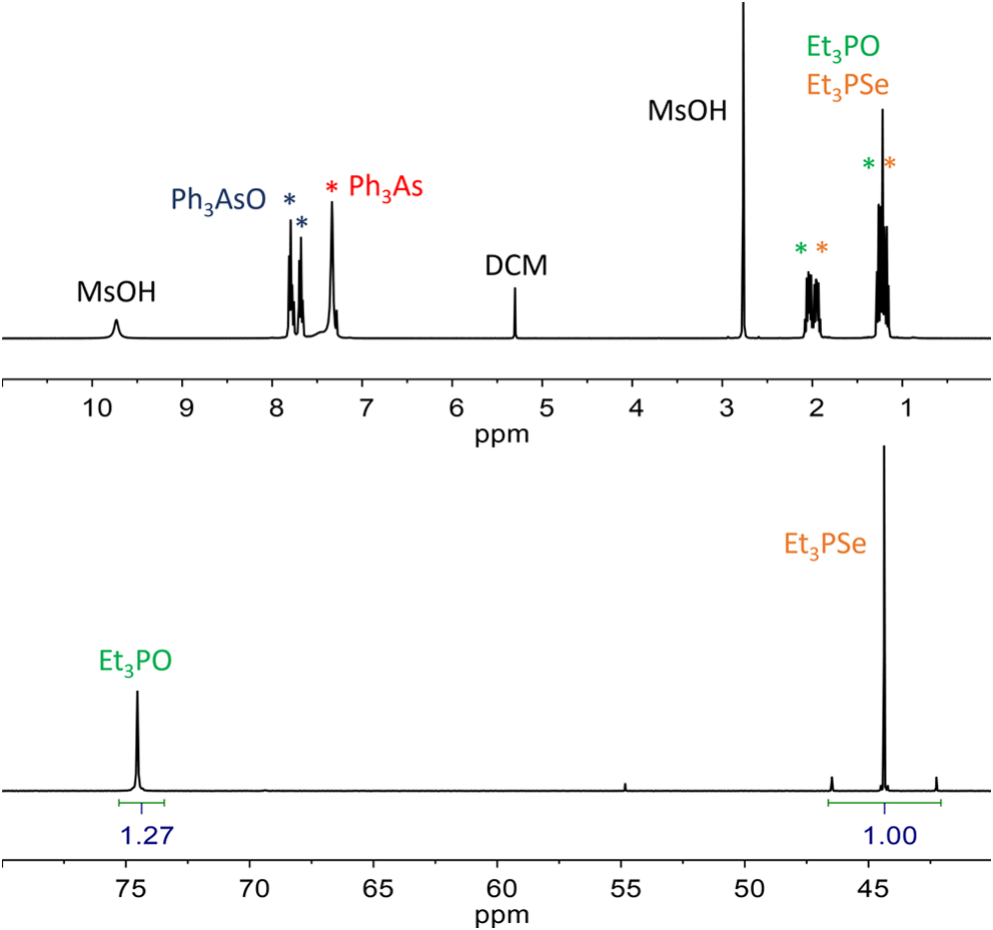
Example of ^1^H (top) and ^31^P{^1^H} (bottom) NMR spectra of the crude reaction mixture (entry 13, Table 1). CDCl_3_, 400 MHz for ^1^H and 162 MHz for ^31^P.

Since triphenylarsane oxide is the most available among the selected arsine oxides, we performed all test chalcogen exchange reactions with it (Table 1). It should be noted that triphenylarsane selenide is thermodynamically unstable and decomposes to triphenylarsane and selenium upon formation.^30^ This, however, does not jeopardize our investigation, since the comparison of the signal of phosphine selenides and phosphine oxides in ^31^P NMR spectra allows us to quantify the conversion (Figure 2, bottom). It is also possible to detect triphenylarsane in the ^1^H NMR spectra since the signals of its CH groups do not overlap with other signals (Figure 2, top).

**Table 1:**
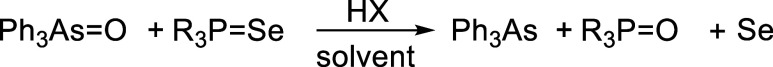
Reaction of Triphenylarsane Oxide with Phosphine Selenides^a^

Run	R	HX	HX, equiv	solvent	*T*, °C	time, h	conversion, %
1	Me	–	–	toluene	110	17	–
2	Me	MsOH	2	toluene	110	17	100
3	Et	MsOH	2	toluene	110	17	100
4	Ph	MsOH	2	toluene	110	17	26
5	Ph	MsOH	2	toluene	110	72	98
6	Et	MsOH	2	dioxane	90	17	100
7	Et	MsOH	2	iPrOH	70	17	100
8	Et	MsOH	2	DCM	25	17	100
9	Et	MsOH	2	DCM	25	1	100
10	Et	TsOH	2	toluene	110	17	100
11	Et	(PhO)_2_POOH	2	toluene	110	17	100
12	Et	Ph_2_POOH	2	toluene	110	17	100
13	Et	4-fluorophenol	2	toluene	110	17	13
14	Et	MsOH	1	DCM	25	17	56
15	Et	MsOH	0.5	DCM	25	17	6
16	Et	MsOH	0.25	DCM	25	17	traces
17	Et	MsOH	0.5	toluene	110	17	97
18	tBu	MsOH	2	toluene	110	17	–

a In a typical experiment, a solution of 0.1 mmol of Ph_3_As in 15 mL of a solvent was used.

We started our experiments with the treatment of Ph_3_AsO with 1 equiv of trimethylphosphane selenide in boiling toluene (to achieve good solubility of the starting material). No reaction occurs even after boiling the reaction mixture for 17 h (Table 1, run 1). The addition of 2 equiv of methanesulfonic acid (MsOH; p*K*_a_ = 1.62 in DMSO, −2.6 in water)^38^ gives complete conversion under the same conditions (run 2). The transition from Me_3_PSe to triethylphosphane selenide does not noticeably affect the reaction outcome in boiling toluene (run 3).

In contrast, the utilization of triphenylphosphane selenide provides only a 26% conversion over 17 h. However, increasing the reaction time to 72 h provides almost complete conversion (runs 4 and 5). The utilization of other solvents with lower boiling temperatures did not affect the reaction (runs 6–8). With Et_3_PSe, the reaction proceeds very fast; thus, a complete conversion can be achieved after stirring the reaction mixture in DCM at room temperature for 1 h (run 9).

Switching to other acids such as *p*-toluenesulfonic acid (TsOH; p*K*_a_ = 0.9 in DMSO, −2.8 in water),^39,40^ diphenylphosphinic acids (Ph_2_POOH, p*K*_a_ = 2.32 in water),^41^ and diphenylphosphoric acid ((PhO)_2_POOH, p*K*_a_ = 3.88 in DMSO)^42^ does not affect the conversion (runs 10–12). Moreover, even such a weak acid as 4-fluorophenol (p*K*_a_ = 9.89 in water)^43^ is still capable of activating a chalcogen exchange reaction; however, it is significantly less effective (run 13). In contrast, reducing the amount of acid results in a dramatic decrease in the reaction rate (runs 14–17).

It is noteworthy that the utilization of sterically hindered tris(*tert*-butyl)phosphane selenide provides no reaction even upon prolonged boiling in toluene in the presence of 2 equiv of acid (run 18).

One can see that the acidity of the acid used is not the determinant factor. Thus, weak diphenylphosphoric acid, which is not capable of a full proton transfer to either arsine oxide or phosphine selenides, acts as effectively as strong TsOH does. Based on this, we can conclude that the reaction is initiated via the hydrogen bond formation between the acid and starting material.

**Figure 3:**
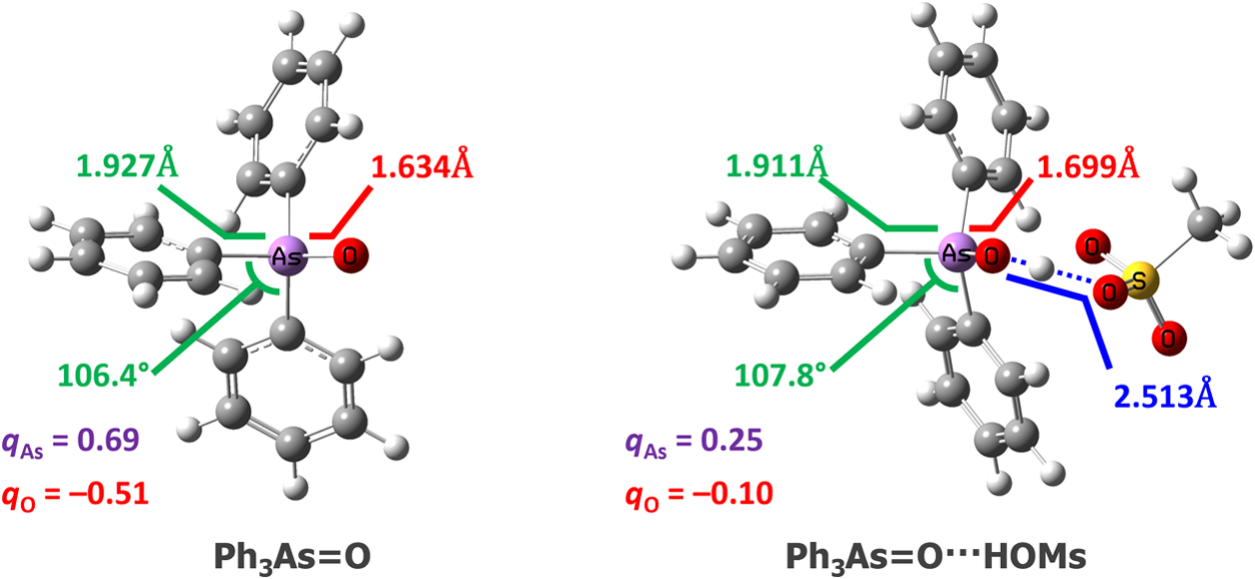
Calculated geometries of triphenylarsane oxide and its complexes with MsOH. Protonation of the As=O moiety results in the elongation of the As–O distance, opening the C–As–C angles, shortening of As–C bonds, and reducing As–O bond polarity.

It is known that triorganinylarsine oxides possess a high proton-accepting ability, which allows the formation of strong H-bonds even with weak donors (see the formation of complexes with H_2_O_2_ above).^34^ According to our calculations, the proton transfer from MsOH to Ph_3_AsO leads to the formation of a short AsO–H-bond (*r*_OH_ = 1.033 Å) with a significant covalent character (ρ ≫ 0, |*V*| ≫ *G*, *H* ≪ 0). One could expect that this protonation leads to the significant polarization of an ^+^As–O^–^ bond, i.e., an additional increase of the positive charge on the arsenic atom upon the formation of the ^+^As–OH moiety. However, even though the protonation results in the increase of the As–O distance by 0.065 Å (Figure 3; see Table S1 in the Supporting Information (SI)) and opening of the C–As–C angles (on average by 1.4°), the polarity of the As–O bond surprisingly becomes smaller: the difference in charges between the As and O atoms shifts from 1.2 to 0.35 after protonation (these values are for the Mulliken population analysis; the results for other atomic charge calculation schemes are provided in the Supporting Information; qualitatively, these results are consistent across all methods).

**Figure 4:**
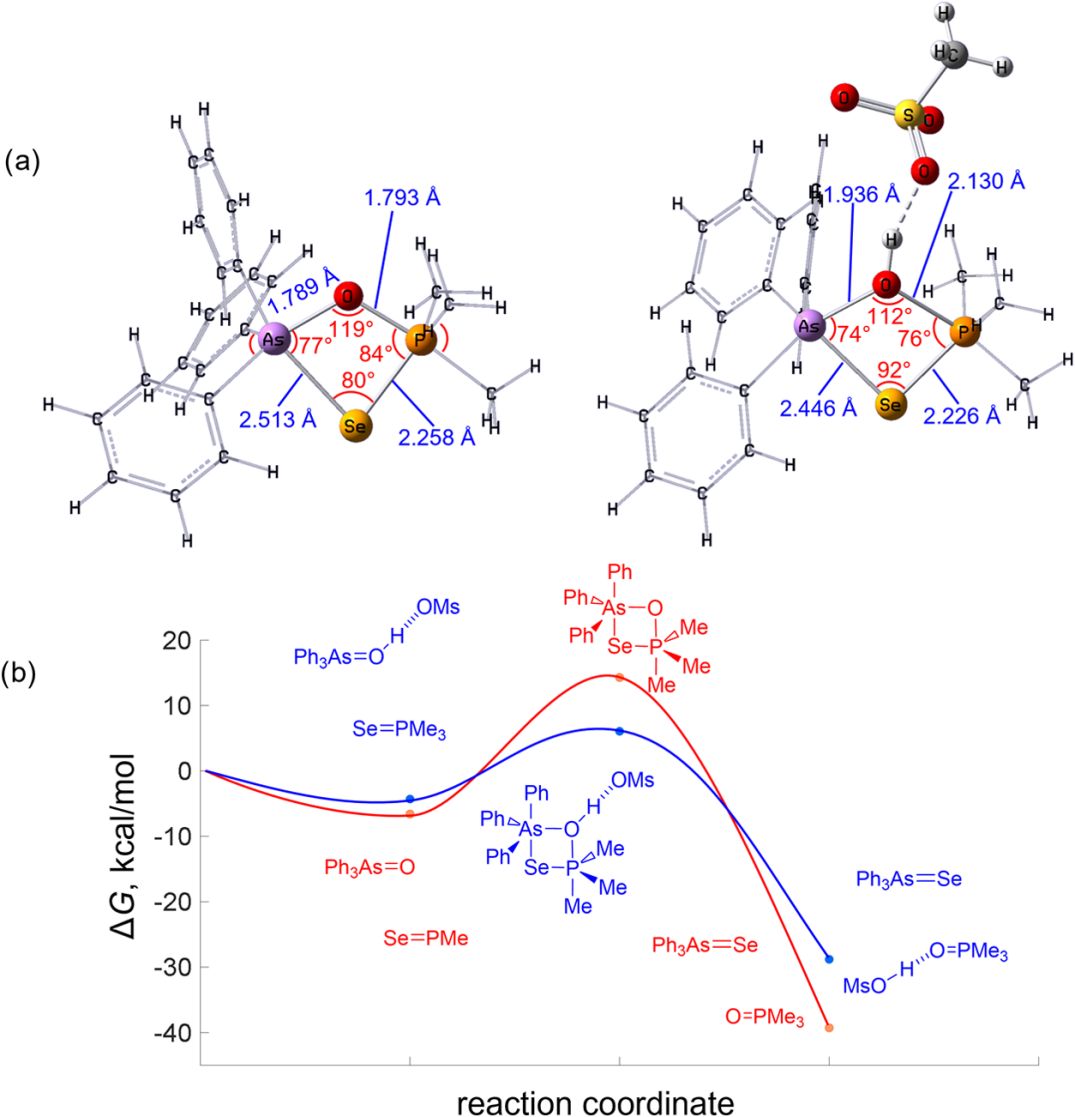
(a) Calculated geometries of cyclic transition states **3** and **4**; some geometric parameters are given in blue and red. (b) Calculated free-energy change upon the chalcogen exchange reaction in the presence (red) and the absence of MsOH (blue).

**Scheme 3:**
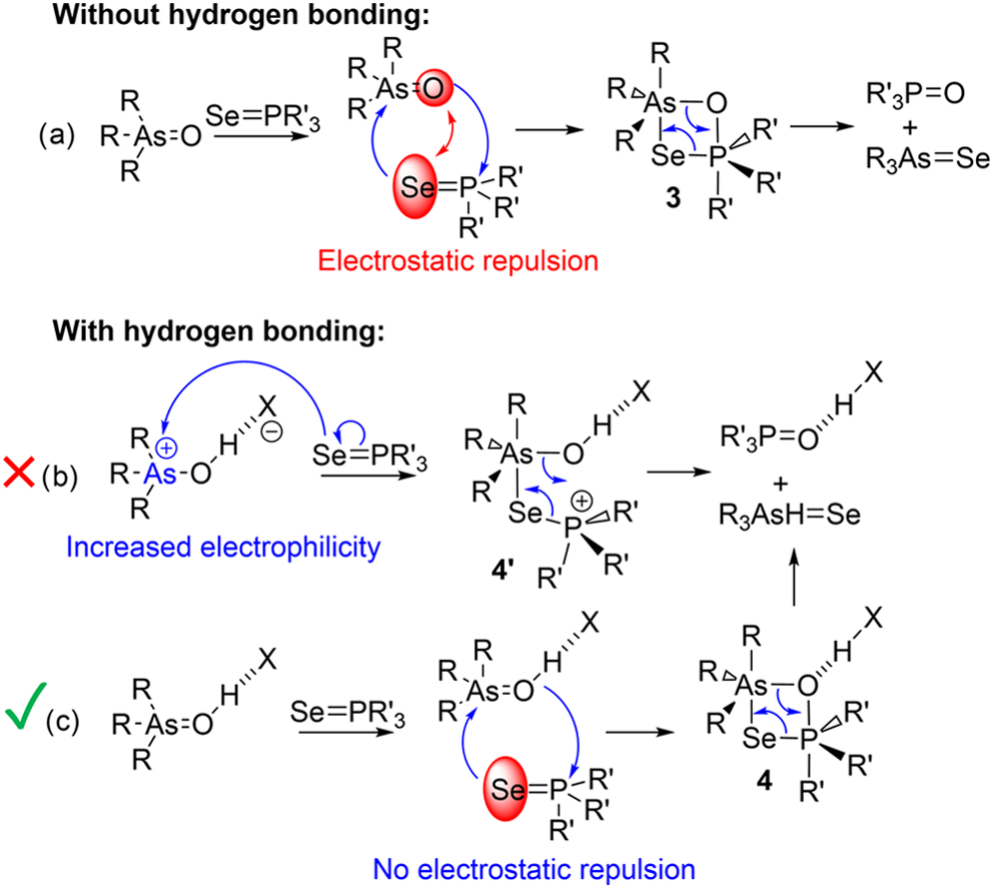
Proposed Mechanisms of Chalcogen Exchange between Phosphine Selenides and Arsine Oxides (a) and the Impact of Hydrogen Bonding on It (b, c)

We believe that the mechanism of the chalcogen exchange between phosphine selenides and arsine oxides is somewhat reminiscent of a Wittig reaction and involves the cyclic transition state **3** (Scheme 3a). The latter is highly strained, and its formation requires overcoming the steric repulsion between substituents at the centers of As and P as well as a significant distortion of the tetrahedral geometry of the reagents (Figure 4). Without an acidic catalyst, this transformation needs to additionally overcome a significant electrostatic repulsion between the P=Se and As=O moieties, bearing a high electron density (see Figure 1). Based on the above-mentioned, we can exclude the activation of arsenic electrophilicity via the protonation of oxygen (Scheme 3b). In contrast, the addition of acid results in the formation of a strong hydrogen-bonded complex with proton transfer toward the oxygen atom. This reduces electrostatic repulsion between the P=Se and As=O moieties, making the formation of cyclic transition state **4** more favorable (Scheme 3c). This is confirmed by the results of quantum chemical modeling of the mechanism of this reaction. Initially, without solvent effects, the activation barrier decreases from Δ*G*^‡^ = 14.2 to Δ*G*^‡^ = 6.0 kcal/mol upon the addition of MsOH. When solvent effects are considered using the IEFPCM model (with dielectric permittivity for toluene ε = 2.3), the activation barrier increases to Δ*G*^‡^ = 16.5 kcal/mol in the absence of MsOH, while it decreases to Δ*G*^‡^ = 5.3 kcal/mol in the presence of MsOH. Despite these quantitative changes, the qualitative conclusion remains unchanged: the presence of MsOH significantly stabilizes the transition state, reducing the activation barrier by approximately 11 kcal/mol in both gas-phase and solvent-inclusive models (Figure 4, bottom). The following opening of the cycle leads to the formation of a free arsine selenide hydrogen-bonded complex of phosphine oxide with acid.

**Figure 5:**
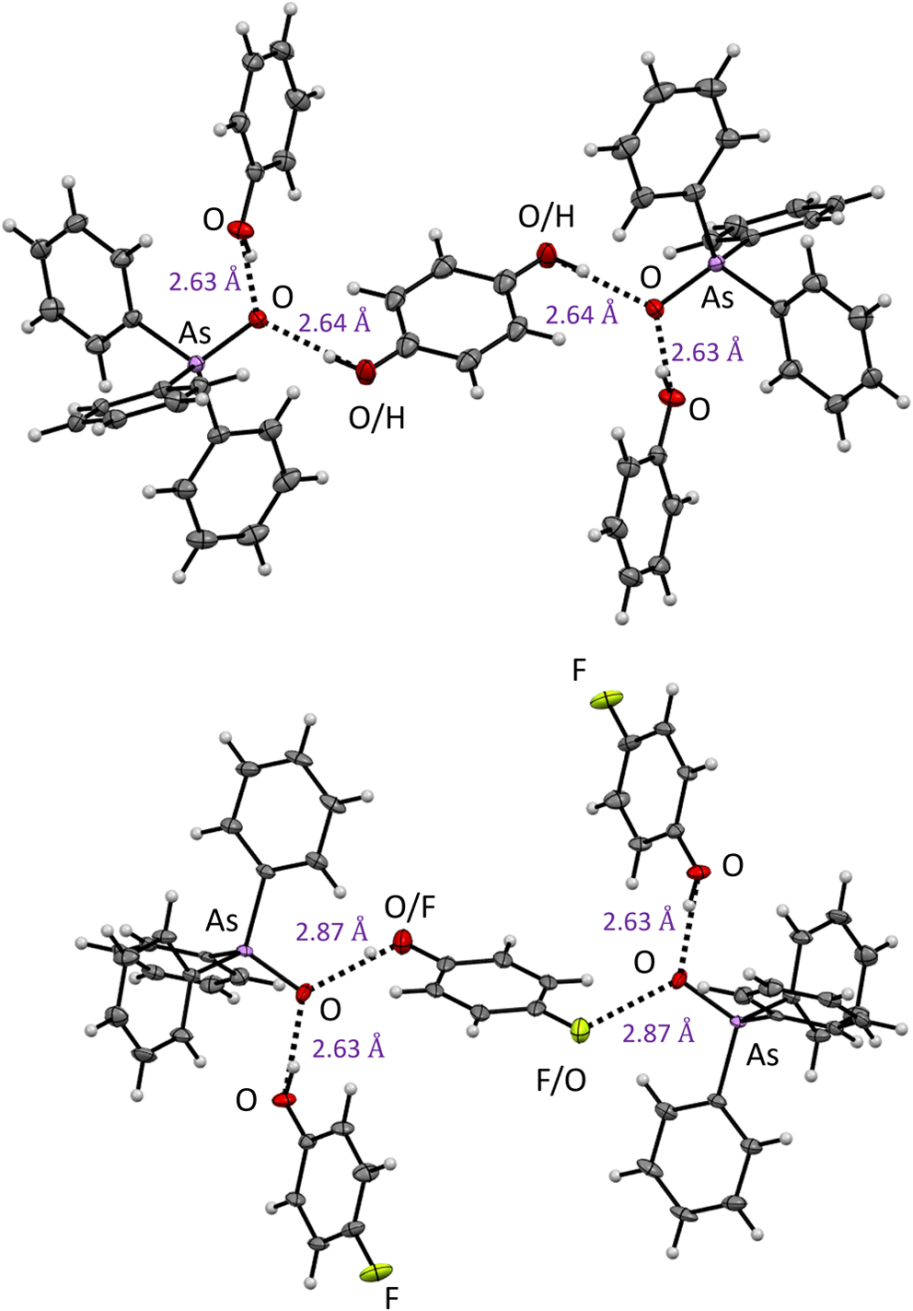
Molecular structures of the H-bonded complexes of triphenylarsane oxide with phenol (top) and 4-fluorophenol (bottom). Disorder of the crystal packing results in the alteration of the orientation of one phenol molecule.

Upon complexation with OH proton donors, IR spectra of phosphine oxides^24^ and arsine oxides (see, for example, ref (37)) undergo changes typical for hydrogen bonding: the appearance of a broader, more intensive, and low-frequency-shifted OH stretching band, as well as a low-frequency shift of a characteristic P=O/As=O stretching band. This is clearly observable in the case of cocrystals, where the complexes in question are the only species^37^ but should be harder to detect if the complexation is an intermediary step along the reaction pathway. It also has to be mentioned that the assignment of bands and the interpretation of IR spectra become more difficult if the As=O band appears near the bands of the proton donor (in its free or complexed form), other reactants, products, and/or solvent. Because of these reasons, we relied on X-ray data, which are virtually free of ambiguity of interpretation. For instance, we have obtained crystal structures for the H-bonded complexes of triphenylarsane and phenols (phenol and 4-fluorophenol), in which two molecules of H-bond donors are simultaneously bound to one oxygen of triphenylarsane, thus proving the capability of arsine oxides to effectively bind even weak acids (Figure 5). Even though no significant proton transfer from phenol to arsine oxide occurs, it is sufficient for the initiation of the reaction (Table 1, entry 13).

The performed kinetic NMR study of the reaction of Ph_3_As=O with Ph_3_P=Se in the presence of 2 equiv of MsOH in CDCl_3_ at 25 °C has demonstrated that the dependence of the conversion rate on the reaction time is almost linear within the first hour of the reaction (see Figure S1 in the SI). Despite the slow transformation in the case of the selected reaction partners, it was not possible to detect any intermediate species (Figure S2 in the SI). We believe that the formation of transition state **4** is a rate-limiting stage, while further chalcogen transfer occurs almost instantly, not allowing for the observation of any intermediates within the NMR time scale.

The discovered transformation was also tested for Et_3_AsO, *n*Bu_3_AsO, and *i*Pr_3_AsO. For these substrates, the formation of corresponding stable arsine selenides with good NMR yields was observed (Table 2). The overall transition from the triphenylarsane oxide to the trialkylarsine oxides expectedly slowed the reaction. We believe that the +I-effect of the alkyl substituents partially suppresses the activation provided by H-bonding. For instance, while Et_3_AsO undergoes a reasonable conversion in DCM at room temperature after 24 h, *n*Bu_3_AsO and *i*Pr_3_AsO containing stronger electron-donating alkyl groups show no reaction under these conditions (runs 2–4). Switching to boiling isopropanol provides a significant conversion increase in the case of Et_3_AsO (runs 5 and 9), and, to a much lesser extent, *i*Pr_3_AsO (runs 7 and 8). The reactivity of *n*Bu_3_AsO is surprisingly not improved under these conditions (run 6). Further increasing the reaction temperature via the transition to boiling dioxane and toluene improves the reaction outcome, even if 0.5 equiv of acid is used (runs 10–15). Overall, even though trialkylarsine oxides are less reactive than Ph_3_AsO, it is possible to achieve excellent conversion by increasing reaction time and/or temperature.

**Table 2:**
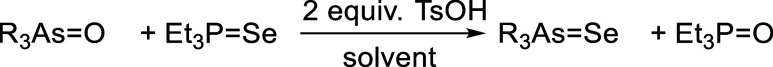
Reaction of Trialkylarsine Oxides with Triethylphosphane Selenide

run	R	solvent	*T*, °C	time, h	conversion, %
1	Et	DCM	25	1	15
2	Et	DCM	25	24	40
3	*n*Bu	DCM	25	24	<1
4	*i*Pr	DCM	25	24	3
5	Et	*i*PrOH	80	24	75
6	*n*Bu	*i*PrOH	80	24	<1
7	*i*Pr	*i*PrOH	80	24	14
8	*i*Pr	*i*PrOH	80	72	80^b^
9	Et	*i*PrOH	80	48	99
10	Et	dioxane	90	24	72
11	*i*Pr	dioxane	90	24	66
12	Et	toluene	110	24	85
13	nBu	toluene	110	24	74
12^a^	Et	toluene	110	24	84
14^a^	*i*Pr	toluene	110	24	97
15	nBu	toluene	110	48	99^c^

a With 0.5 equiv of TsOH.

b 23% isolated yield.

c 27% isolated yield.

Due to the high sensitivity of arsine selenides to oxygen, their isolation from the reaction mixture is a challenging task: chromatography cannot be used, and we must rely on the solubility differences between arsine selenides and phosphine oxides, which allows one to “wash” the main products from byproducts. Nevertheless, it was possible to obtain pure nBu_3_As=Se and iPr_3_As=Se via extracting them from the crude reaction mixture with hot hexane. Unfortunately, the utilization of a similar approach for the isolation of Et_3_AsSe was unsuccessful due to the similar solubility of the desired product and Et_3_P=O.

## Conclusions

In summary, we demonstrated an easy hydrogen-bond-assisted chalcogen exchange reaction between phosphine selenides and arsine oxides under mild conditions. The reaction can be carried out in various solvents (DCM, *i*PrOH, dioxane, toluene) with comparable outcomes. The transformation is dramatically facilitated by any nonaqueous acid. It is possible to perform the transformation with a nonstoichiometric amount of acid; however, harsher reaction conditions and/or a prolonged reaction time are required to achieve a good conversion. The discovered reaction is suitable for various arsine oxides and phosphine selenides; however, the utilization of reagents bearing bulky substituents (*t*Bu or *i*Pr) significantly hampers the reaction performance. Even though it was not possible to achieve a good isolated yield, the developed concept allows the preparation of sterically hindered arsine selenides (such as *i*Pr_3_As=Se), which are inaccessible via direct oxidation of corresponding arsines with selenium. Using quantum chemical calculations, we have shown that the reaction’s mechanism involves the formation of a four-membered cyclic intermediate, requiring overcoming steric and electrostatic repulsion, as well as a significant distortion of the tetrahedral geometry of the reagents. The involvement of an As=O fragment in hydrogen bonding does not increase the electrophilicity of the arsenic atom: surprisingly, the polarity of the As–O bond becomes smaller upon the formation of a H-bonded complex with acid. At the same time, the formation of a hydrogen bond reduces the electrostatic repulsion between the P=Se and As=O moieties, making the formation of a cyclic intermediate more favorable. This important finding opens a new perspective for the utilization of hydrogen bonding for the simple synthesis of pnictine chalcogenides via a chalcogen exchange reaction.

## Experimental Part

### General

Toluene, dioxane, diethyl ether, and THF were dried over sodium/benzophenone. Dichloromethane was dried over 4 Å molecular sieves. Isopropanol was dried over calcium hydride.

Liquid-state NMR experiments were performed using a Bruker Avance III NMR spectrometer (400 MHz for ^1^H, 101 MHz for ^13^C, 162 MHz for ^31^P, and 76 MHz for ^77^Se) at the Center for Magnetic Resonance, St. Petersburg State University Research Park. Chemical shifts are referenced to TMS for ^1^H and ^13^C, to phosphoric acid for ^31^P, and to Me_2_Se for ^77^Se.

HR-ESI mass spectra were obtained on a BRUKER maXis spectrometer equipped with an electrospray ionization (ESI) source; methanol was used as the solvent at the Chemical Analysis and Materials Research Centre, St. Petersburg State University Research Park. The instrument was operated in positive mode using a *m*/*z* range of 50–1200. The capillary voltage of the ion source was set at 4000 V. The nebulizer gas pressure was 1.0 bar, and the drying gas flow was set to 4.0 L/min.

### Computations

Computational resources were provided by the Computer Center of Saint Petersburg University Research Park (http://www.cc.spbu.ru/). The calculations were carried out using the Gaussian16 rev A.03 software package.^44^ Geometry optimizations (with standard convergence criteria for forces and displacements) and vibrational harmonic frequencies calculations were performed at the PW6B96-D3/def2-TZVPD level of theory. The Grimme dispersion correction D3 with zero damping was included.^45^ All structures were checked for the absence of imaginary vibrational frequencies except for transition states 3, 4, and 4′, which were optimized to a first-order saddle point on the potential energy surface. Thermodynamic parameters were obtained using a standard Gaussian thermochemistry module and 1 atm, 298 K conditions.

The MultiWFN^46^ program was used for calculating the surfaces of electron density, electron localization function,^47^ and molecular electrostatic potential.^48^ The visualization was performed by using GaussView and Matlab R2021b.

Single crystals of Ph_3_AsO complexes with phenol and 4-fluorophenol were grown by the slow evaporation of a chloroform solution at +25 °C. The single-crystal X-ray diffraction data were collected using the SuperNova diffractometer equipped with a HyPix-3000 detector and a microfocus Cu Ka radiation source (λ = 1.54184 Å) at temperature *T* = 100 (2) or 120 K at the Centre for X-ray Diffraction Studies, St. Petersburg State University Research Park. Using Olex216, the structure was solved with the SHELXT^49^ structure solution program using Intrinsic Phasing and refined with the SHELXL^50^ refinement package using least-squares minimization.

### Synthetic Procedures

Caution! Arsenic compounds are highly toxic. All operations must be performed in a fume hood with excellent ventilation.

#### Arsenic(III) Iodide

An excess of hot (100 °C) 54% aqueous hydrogen iodide solution (5 mL) was added to finely ground sodium *ortho*-arsenite Na_3_AsO_3_ (0.96 g, 5 mmol) under constant stirring at 100 °C. After 1 min, the hot reaction mixture with the brown suspension was filtered quickly (to prevent hydrolysis of the product). The precipitate was washed with small portions of cold diethyl ether until the whole substance became orange. The obtained substance was extracted with chloroform using a Soxhlet extractor (3 h). The dark red solution was cooled to −25 °C, and after 12 h, the orange crystals formed were filtered and dried under vacuum, mp 145–146 °C and yield 63% (1.415 g, 3.1 mmol).

#### Synthesis of Phosphine Selenides R_3_PSe

Tris(*tert*-butyl)phosphane selenide (*t*Bu_3_PSe) and triphenylphosphane selenide (Ph_3_PSe) were synthesized according to the standard methodology described in the literature^25,51,52^ from commercially available phosphines R_3_P (where R = *t*Bu and Ph). For this, phosphine (2.0 mmol) was mixed with gray selenium (130 mg, 3.0 mmol) in dry toluene (30 mL). The mixture was boiled for 5 h and filtered. The solvent was removed, and the product was purified by recrystallization in toluene. Triphenylphosphane selenide, yield 615 mg (90%), white solid. ^1^H NMR (400 MHz, CDCl_3_) δ = 7.80–7.70 (m, 6H), 7.57–7.41 (m, 9H). ^13^C{^1^H} NMR (101 MHz, CDCl_3_) δ = 132.7 (d, *J* = 10.8), 131.82 (d, *J* = 76.8), 131.6 (d, *J* = 3.1), 128.5 (d, *J* = 12.5). ^31^P{^1^H} NMR (162 MHz, CDCl_3_) δ = 35.3. ^77^Se{^1^H} NMR (76 MHz, CDCl_3_) δ = −267 (d, *J* = 730.8).

Tris(*tert*-butyl)phosphane selenide, yield 480 mg (85%), white solid. ^1^H NMR (400 MHz, CDCl_3_) δ = 1.54 (d, *J* = 14.0, 27H). ^13^C{^1^H} NMR (101 MHz, CDCl_3_) δ = 41.2 (d, *J* = 26.1), 30.5. ^31^P{^1^H} NMR (162 MHz, CDCl_3_) δ = 92.7. ^77^Se{^1^H} NMR (76 MHz, CDCl_3_) δ = −423. (d, *J* = 687.5).

Trimethylphosphane selenide (Me_3_PSe) and triethylphosphane selenide (Et_3_PSe) were prepared using our previously reported one-pot method.^25^ For this, a commercial solution of the corresponding organolithium reagent RLi (3.6 mmol, where R = Me and Et) in diethyl ether was added dropwise to the cooled 0 °C solution of phosphorus trichloride PCl_3_ (0.1 mL, 1.1 mmol) in dry toluene (30 mL) under an argon atmosphere. The reaction mixture was left overnight at room temperature with stirring. Gray selenium (135 mg, 1.7 mmol) was added; the mixture was refluxed for 5 h and filtered. The solvent was removed, and the product was purified by recrystallization in toluene.

Trimethylphosphane selenide, yield 110 mg (64%), white solid. ^1^H NMR (400 MHz, CDCl_3_) δ = 1.98 (d, *J* = 13.3, 9H). ^13^C{^1^H} NMR (101 MHz, CDCl_3_) δ = 23.0 (d, *J* = 49.1). ^31^P{^1^H} NMR (162 MHz, CDCl_3_) δ = 8.7. ^77^Se{^1^H} NMR (95 MHz, CDCl_3_) δ = −248 (d, *J* = 689.6 Hz).

Triethylphosphane selenide, yield 110 mg (72%), white solid. ^1^H NMR (400 MHz, CDCl_3_) δ = 1.95 (dq, *J* = 11.5, 7.6, 6H), 1.20 (dt, *J* = 18.9, 7.6, 9H). ^13^C{^1^H} NMR (101 MHz, CDCl_3_) δ = 22.8 (d, *J* = 45.0), 7.2 (d, *J* = 4.5). ^31^P{^1^H} NMR (162 MHz, CDCl_3_) δ = 44.2. ^77^Se{^1^H} NMR (95 MHz, CDCl_3_) δ = −413 (d, *J* = 685.7).

#### Synthesis of Arsine Oxides R_3_AsO

Triphenylarsane oxide (Ph_3_AsO) was prepared by the oxidation of commercially available triphenylarsane with hydrogen peroxide, as previously shown for other arsines.^36,37^ For this, triphenylarsane (918 mg, 3.0 mmol) was dissolved in acetonitrile (50 mL) and 30% aqueous solution of hydrogen peroxide (0.46 mL, 4.5 mmol) was added. The reaction mixture was stirred at room temperature overnight. The solvent was removed, and the residue was dissolved in dry toluene (50 mL). Activated 4 Å molecular sieves (2 g) were added, and the mixture was refluxed for 2 h to break the H-bonded adduct of triphenylarsane oxide with hydrogen peroxide (Ph_3_AsO·H_2_O_2_). The mixture was filtered, and the solvent was removed to give pure Ph_3_AsO as a white solid, yield = 870 mg (91%).

^1^H NMR (400 MHz, CDCl_3_) δ = 7.76–7.70 (m, 6H), 7.60–7.54 (m, 3H), 7.54–7.48 (m, 6H). ^13^C NMR (101 MHz, CDCl_3_) δ = 133.0, 132.1, 131.5, and 129.3.

Aliphatic arsine oxides R_3_AsO (R = Et, *i*Pr, *n*Bu) were prepared using the one-pot method. For this, arsenic(III) iodide (500 mg, 1.1 mmol) was suspended in dry diethyl ether (30 mL), cooled to 0 °C, and the solution of the corresponding organomagnesium (R = Et and *i*Pr) or organolithium (R = *n*Bu) reagent (3.6 mmol) in dry diethyl ether (R = *n*Bu) or THF (R = Et and *i*Pr) was added dropwise. The reaction mixture was left overnight at room temperature with stirring. A 30% aqueous solution of hydrogen peroxide (0.12 mL, 1.2 mmol) was added to the obtained mixture. The reaction mixture was stirred at room temperature overnight and then filtered. The solvent was removed, and the residue was dissolved in dry toluene (30 mL). Activated 4 Å molecular sieves (1 g) were added, and the mixture was refluxed for 2 h. The mixture was filtered, and the solvent was removed. The residue was treated with DCM (3 × 20 mL) and filtered; the solvent was evaporated to dryness to give pure corresponding arsine oxide. The developed one-pot approach allows us to avoid operations with isolated volatile and toxic trialkylarsines.

Triethylarsane oxide, yield 150 mg (77%), amorphous yellowish solid. ^1^H NMR (400 MHz, CD_3_OH) δ = 2.14 (q, *J* = 7.8, 6H, CH_2_), 1.31 (t, *J* = 7.8, 9H, CH_3_). ^13^C NMR (101 MHz, CD_3_OH) δ = 19.9 (CH_2_), 5.4 (CH_3_). HR-ESi MS: *m*/*z* 179.0409. Calculated for C_6_H_16_OAs [M + H]^+^ = 179.0417.

Tri(*n*-butyl)arsane oxide, yield 147 mg (51%), amorphous yellowish solid. ^1^H NMR (400 MHz, CD_3_OH) δ = 2.12–2.01 (m, 6H, ^1^CH_2_), 1.70–1.57 (m, 6H, ^2^CH_2_), 1.47 (dq, *J* = 13.9, 7.0, 6H, ^3^CH_2_), 0.97 (t, *J* = 7.3, 9H, CH_3_). ^13^C{^1^H} NMR (101 MHz, CD_3_OH) δ = 28.4 (^1^CH_2_), 25.1 (^2^CH_2_), 25.0 (^3^CH_2_), 13.7 (CH_3_). HR-ESi MS: *m*/*z* 263.1344. Calculated for C_12_H_28_OAs [M + H]^+^ = 263.1356.

Tri(*iso*-propyl)arsane oxide, yield 148 mg (67%), amorphous yellowish solid. ^1^H NMR (400 MHz, CD_3_OH) δ = 2.76 (hept, *J* = 7.3, 3H, CH), 1.41 (d, *J* = 7.3, 18H, CH_3_). ^13^C NMR (101 MHz, CD_3_OH) δ = 29.3 (CH), 16.1 (CH_3_). HR-ESI MS: *m*/*z*, 221.0883. Calculated for C_9_H_22_OAs [M + H]^+^ = 221.0887.

#### Synthesis of Arsine Selenides R_3_AsSe

The attempt to prepare aliphatic arsine selenides R_3_AsSe (R = *n*Bu and *i*Pr) was performed via a similar one-pot method described above for arsine oxides. After completion of the reaction of arsenic iodide with organomagnesium or an organolithium reagent, gray selenium (133 mg, 1.7 mmol) was added, and the mixture was refluxed for 5 h and filtered. The solvent was removed to give the corresponding arsine selenides.

Tri(*n*-butyl)arsane selenide: yield 250 mg (70%), yellow oil. ^1^H NMR (400 MHz, CD_3_OH) δ = 2.13 (t, *J* = 8.3, 6h, ^1^CH_2_), 1.57 (m, 6H, ^2^CH_2_), 1.45 (sext, *J* = 7.3, 6H, ^3^CH_2_), 0.94 (t, *J* = 7.3, 9H, CH_3_). ^13^C{^1^H} NMR (101 MHz, CD_3_OH) δ = 30.2 (^1^CH_2_), 26.7 (^2^CH_2_), 24.4 (^3^CH_2_), 13.8 (CH_3_). ^77^Se{^1^H} NMR (76 MHz, CD_3_OH) δ = – 312. HR-ESI MS: *m*/*z*, 327.0566. Calculated for C_12_H_28_AsSe [M + H]^+^ = 327.0567.

### General Procedure for Chalcogen Exchange Reactions

Arsine oxide (0.1 mmol) was dissolved in an appropriate solvent (15 mL) in a 50 mL round bottomed flask with a condenser. An equimolar amount of phosphine selenide was added. A certain amount of acid was added. The reaction mixture was kept under argon at a certain temperature for a certain amount of time while stirring. The solvent was removed; the residue was dissolved in CDCl_3_ and subjected to the NMR study. The relative ratio of products was determined by comparing the integral intensities of the phosphine selenide and phosphine oxide signals in ^31^P NMR spectra.

Tri(*n*-butyl)arsane selenide was obtained via the reaction of *n*Bu_3_AsO (18 mg, 0.07 mmol) with Et_3_PSe (13 mg, 0.07 mmol) in the presence of TsOH (23 mg, 0.14 mmol) in toluene (110 °C, 48 h). The product was isolated by washing the crude reaction mixture with hot hexane. The hexane solution was decanted from the precipitate, and the solvent was evaporated in vacuum. Yield 6 mg (27%), yellow oil. ^1^H NMR (400 MHz, CDCl_3_) δ = 2.18 (t, *J* = 7.8, 6h, ^1^CH_2_), 1.60 (m, 6H, ^2^CH_2_), 1.43 (sext, *J* = 7.3, 6H, ^3^CH_2_), 0.94 (t, *J* = 7.3, 9H, CH_3_). ^13^C{^1^H} NMR (101 MHz, CDCl_3_) δ = 29.9 (^1^CH_2_), 29.8 (^2^CH_2_), 24.5 (^3^CH_2_), 13.8 (CH_3_). HR-ESI MS: *m*/*z*, 327.0565. Calculated for C_12_H_28_AsSe [M + H]^+^ = 327.0567.

Tri(*iso*-propyl)arsane selenide was obtained via the reaction of *i*Pr_3_AsO (17 mg, 0.08 mmol) with Et_3_PSe (15 mg, 0.08 mmol) in the presence of TsOH (26 mg, 0.16 mmol) in iPrOH (80 °C, 72 h). The product was isolated via washing the crude reaction mixture with hot hexane. The hexane solution was decanted from the precipitate, and the solvent was evaporated under vacuum. Yield 5 mg (23%), amorphous yellowish solid. ^1^H NMR (400 MHz, CDCl_3_) δ = 2.45 (hept, *J* = 7.2, 3H, CH), 1.33 (d, *J* = 7.2 Hz, 18H, CH_3_). ^13^C{^1^H} NMR (101 MHz, CDCl_3_) δ = ppm: 29.6 (CH), 19.0 (CH_3_). ^77^Se{^1^H} NMR (76 MHz, CD_3_OH) δ = −348. HR-ESI MS: *m*/*z*, 285.0110. Calculated for C_9_H_22_AsSe [M + H]^+^ = 285.0098.

Triethylarsane selenide was obtained via the reaction of Et_3_AsO (14 mg, 0.08 mmol) with Et_3_PSe (15 mg, 0.08 mmol) in the presence of TsOH (26 mg, 0.16 mmol) in *i*PrOH (80 °C, 24 h). The product was isolated via washing the crude reaction mixture with hot hexane. The hexane solution was decanted from the precipitate, and the solvent was evaporated under vacuum. The residue (5 mg) is an amorphous yellowish solid, consisting of triethylarsane selenide and unreacted triethylphosphane selenide. ^1^H NMR (400 MHz, CDCl_3_) δ = 2.05 (q, *J* = 7.7 Hz, 6H, CH_2_), 1.24–1.30 (m, 9H, CH_3_).

## Data Availability

The data underlying this study are available in the published article and its Supporting Information.

## Supporting Information Available

Computational details, NMR spectra, and X-ray data for the obtained compounds (PDF)
