# Supplementary material for: Hydrogen-Bond-Assisted Chalcogen Transfer between Phosphine Selenides and Arsine Oxides
Source: Inorg Chem. 2025 May 5;64(19):9447–56. doi: 10.1021/acs.inorgchem.4c05433 (PMC12093297; doi:10.1021/acs.inorgchem.4c05433)
Supplement: Supplementary file 1 — ic4c05433_si_001.pdf [file ic4c05433_si_001.pdf]

Supporting information for

Hydrogen-bond-assisted Chalcogen Transfer Between Phosphine Selenides and Arsine Oxides.

Danil V. Krutin,[1] Semyon V. Tsybulin,[1] Valeriya V. Mulloyarova,[1] Elena Yu. Tupikina,[1] Peter M. Tolstoy,[1] and Alexander S. Antonov*[2]

[1]St. Petersburg State University, Institute of Chemistry, Universitetskii pr. 26, 198504 St. Petersburg, Russian Federation.

[2]University of Regensburg, Institute of Organic Chemistry, D-93053 Regensburg, Germany.

e-mail: Alexander.Antonov@chemie.uni-regensburg.de

Table of Contents

Reaction NMR monitoring S2
Topology analysis S4
Population analysis S5
Copies of ^{1}H, ^{13}C, ^{31}P and ^{77}Se NMR spectra of phosphine selenides R_3PSe S6
Copies of ^{1}H and ^{13}C NMR spectra of arsine oxides R_3AsO S12
Copies of ^{1}H, ^{13}C and ^{77}Se NMR spectra of arsine selenides R_3AsSe S16
X-ray studies S21

Reaction NMR monitoring

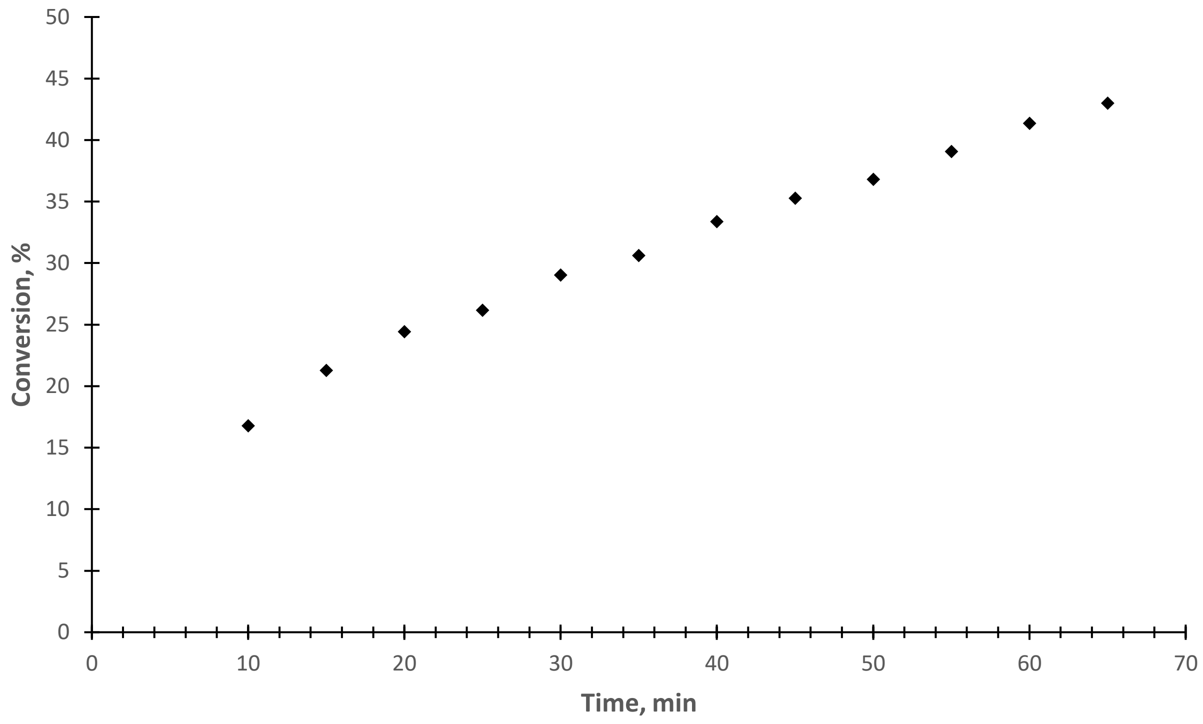

Figure S1. Dependence of the conversion of Et_3PSe into Et_3PO (%) in the presence of Ph_3AsO and 2 equiv. of MsOH on the reaction time (min). Determined based on the ratio of integral intensities.

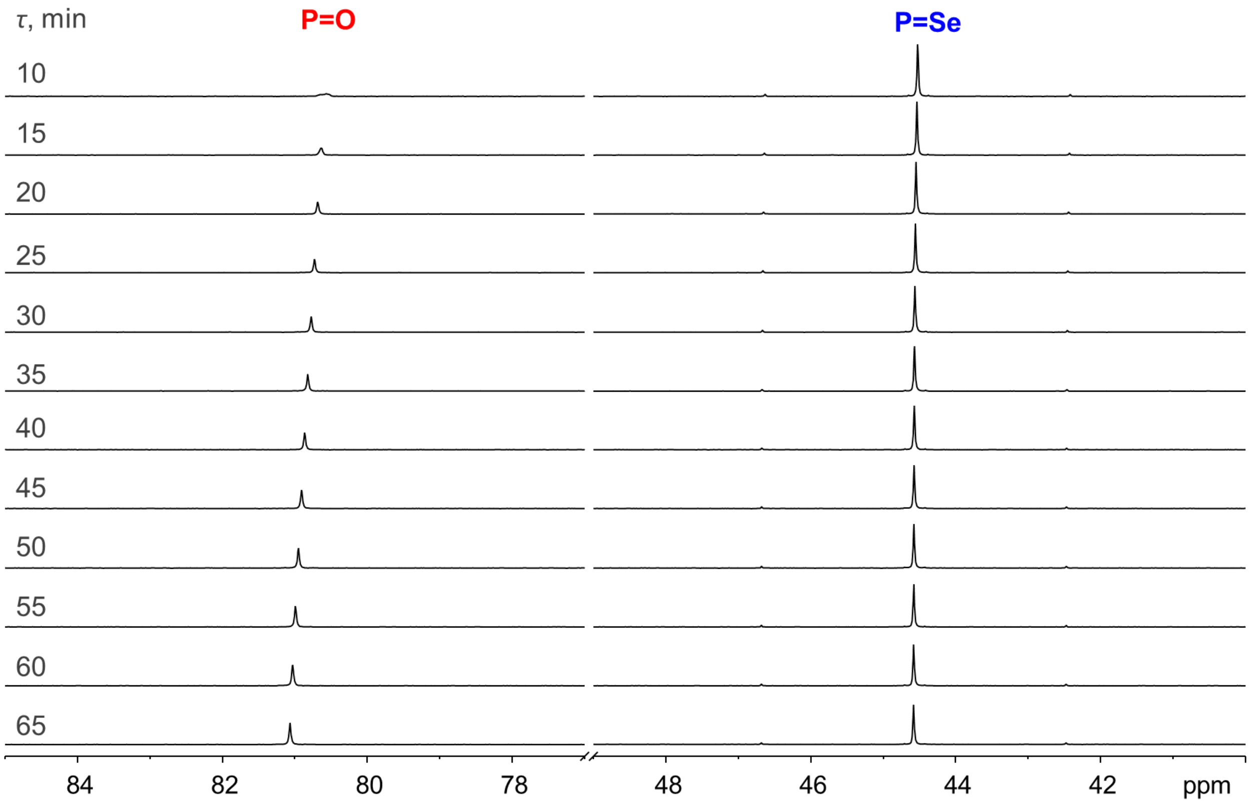

Figure S2. ^{31}P NMR spectra recorded at certain time intervals for reaction mixture containing equimolar amounts of Et_3PSe and Ph_3AsO and 2 equiv. of MsOH (162 MHz, RT, $CDCl_3$)

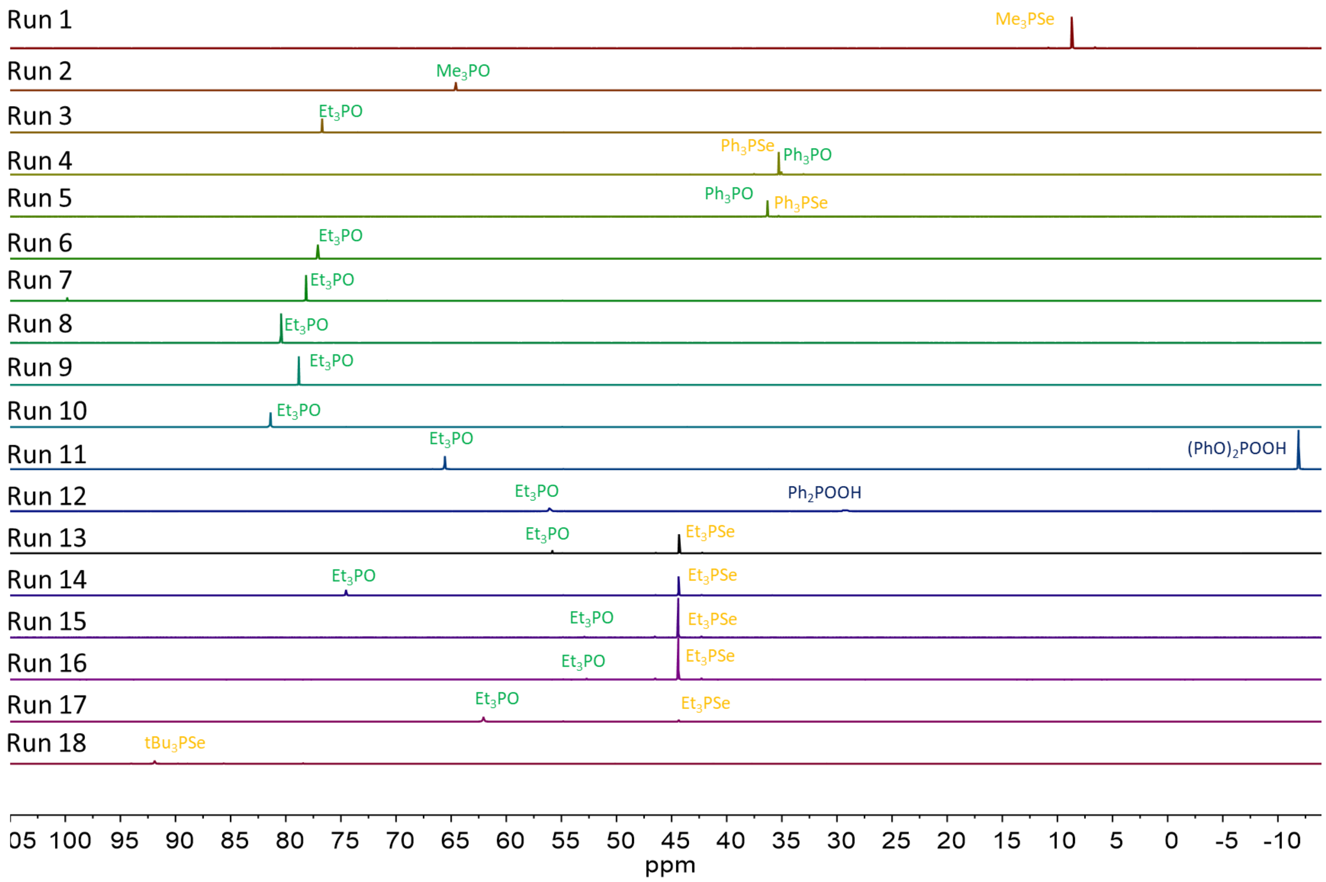

Figure S3. ^{31}P NMR spectra of the reaction mixtures presented in Table 1 (162 MHz, RT, $CDCl_3$).

Topology analysis

Table S1. Geometry parameters, electron density values *ρ* and its Laplacian $\nabla^2\rho$, kinetic *G* and potential *V* energy densities of electrons at electron density critical points of type (3, –1) for bonds of triphenylarsane oxide and its associate with methanesulfonic acid

System	As=O bond					
	R, Å	*ρ*, $Bohr^{-3}$	$\nabla^2\rho$, $Bohr^{-5}$	*G*, Hartree·$Bohr^{-3}$	*V*, Hartree·$Bohr^{-3}$	*H*, Hartree·$Bohr^{-3}$
Ph_3AsO	1.634	0.2151	0.8093	0.3363	–0.4702	–0.1339
$Ph_3AsOH\cdots OMs$	1.699	0.1858	0.6281	0.2621	–0.3671	–0.1050
System	As–C bond					
	R, Å	*ρ*, $Bohr^{-3}$	$\nabla^2\rho$, $Bohr^{-5}$	*G*, Hartree·$Bohr^{-3}$	*V*, Hartree·$Bohr^{-3}$	*H*, Hartree·$Bohr^{-3}$
Ph_3AsO	1.927	0.1504	–0.0896	0.0750	–0.1724	–0.0974
$Ph_3AsOH\cdots OMs$	1.911	0.1565	–0.1275	0.0726	–0.1771	–0.1045
System	O···H bond					
	R, Å	*ρ*, $Bohr^{-3}$	$\nabla^2\rho$, $Bohr^{-5}$	*G*, Hartree·$Bohr^{-3}$	*V*, Hartree·$Bohr^{-3}$	*H*, Hartree·$Bohr^{-3}$
$Ph_3AsOH\cdots OMs$	1.033	0.2746	–1.6768	0.0718	–0.5627	–0.4909
System	H–O bond					
	R, Å	*ρ*, $Bohr^{-3}$	$\nabla^2\rho$, $Bohr^{-5}$	*G*, Hartree·$Bohr^{-3}$	*V*, Hartree·$Bohr^{-3}$	*H*, Hartree·$Bohr^{-3}$
$Ph_3AsOH\cdots OMs$	1.482	0.0784	0.1299	0.0613	–0.0901	–0.0288

System	Angles, °		
	C–As–C	*O···H–O*	*As=O···H*
Ph_3AsO	106.4	-	-
$Ph_3AsOH\cdots OMs$	107.8	176.0	115.6

Population analysis

Table S2. Charges on atoms As and O for Ph_3AsO and $Ph_3AsOH\cdots OMs$ calculated using different population analysis schemes.

		Ph_3AsO	$Ph_3AsOH\cdots OMs$
VDD	q_{As}, e	0.49	0.52
	q_O, e	–0.53	–0.10
	q_{As} + |q_O|, e	1.02	0.62
CHELPG	q_{As}, e	0.63	0.53
	q_O, e	–0.66	–0.51
	q_{As} + |q_O|, e	1.29	1.04
ADCH	q_{As}, e	0.69	0.25
	q_O, e	–0.51	–0.34
	q_{As} + |q_O|, e	1.20	0.59
Becke	q_{As}, e	1.93	–0.57
	q_O, e	–0.54	–0.48
	q_{As} + |q_O|, e	2.47	–0.09
MK	q_{As}, e	0.38	0.10
	q_O, e	–0.60	–0.43
	q_{As} + |q_O|, e	0.98	0.53

Copies of ^{1}H, ^{13}C, ^{31}P and ^{77}Se NMR spectra of phosphine selenides R_3PSe

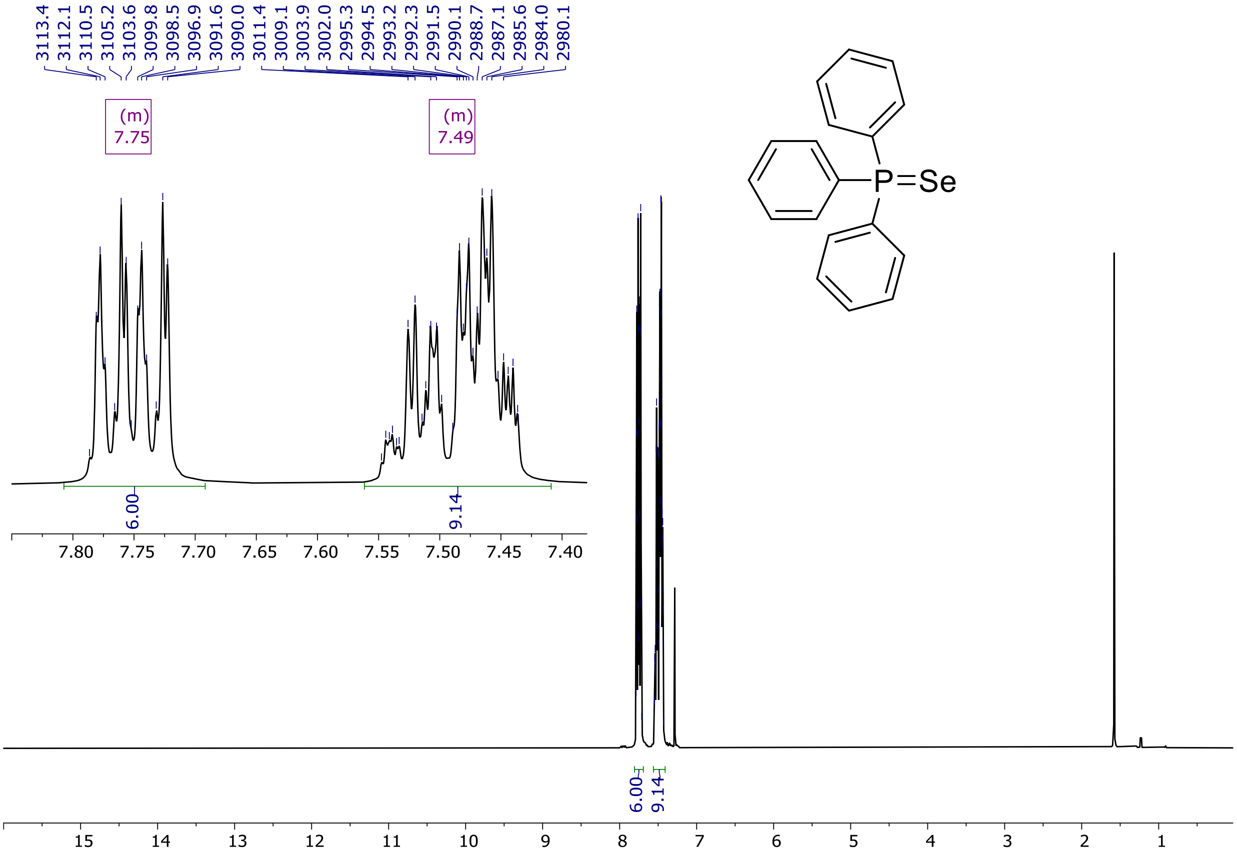

Figure S4. ^{1}H NMR spectrum of triphenylphosphane selenide (400 MHz, RT, $CDCl_3$)

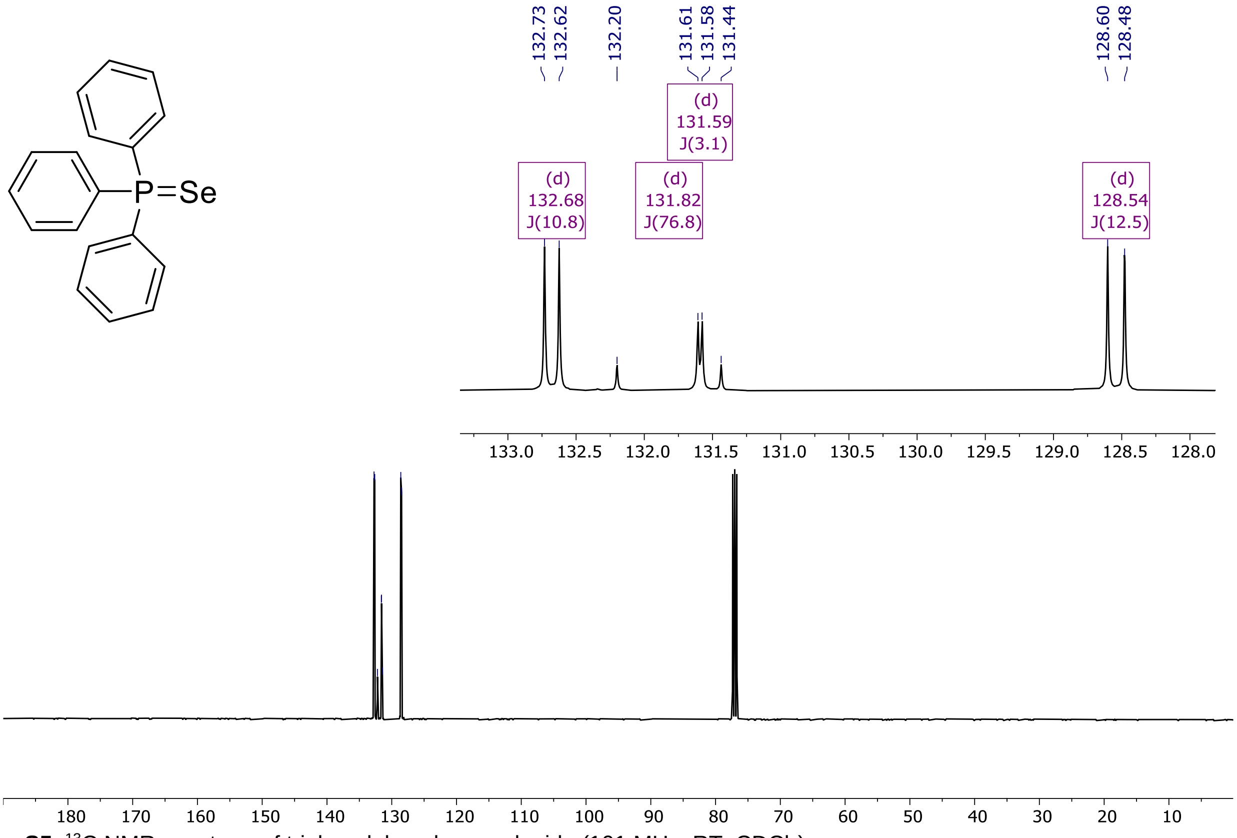

Figure S5. ^{13}C NMR spectrum of triphenylphosphane selenide (101 MHz, RT, $CDCl_3$)

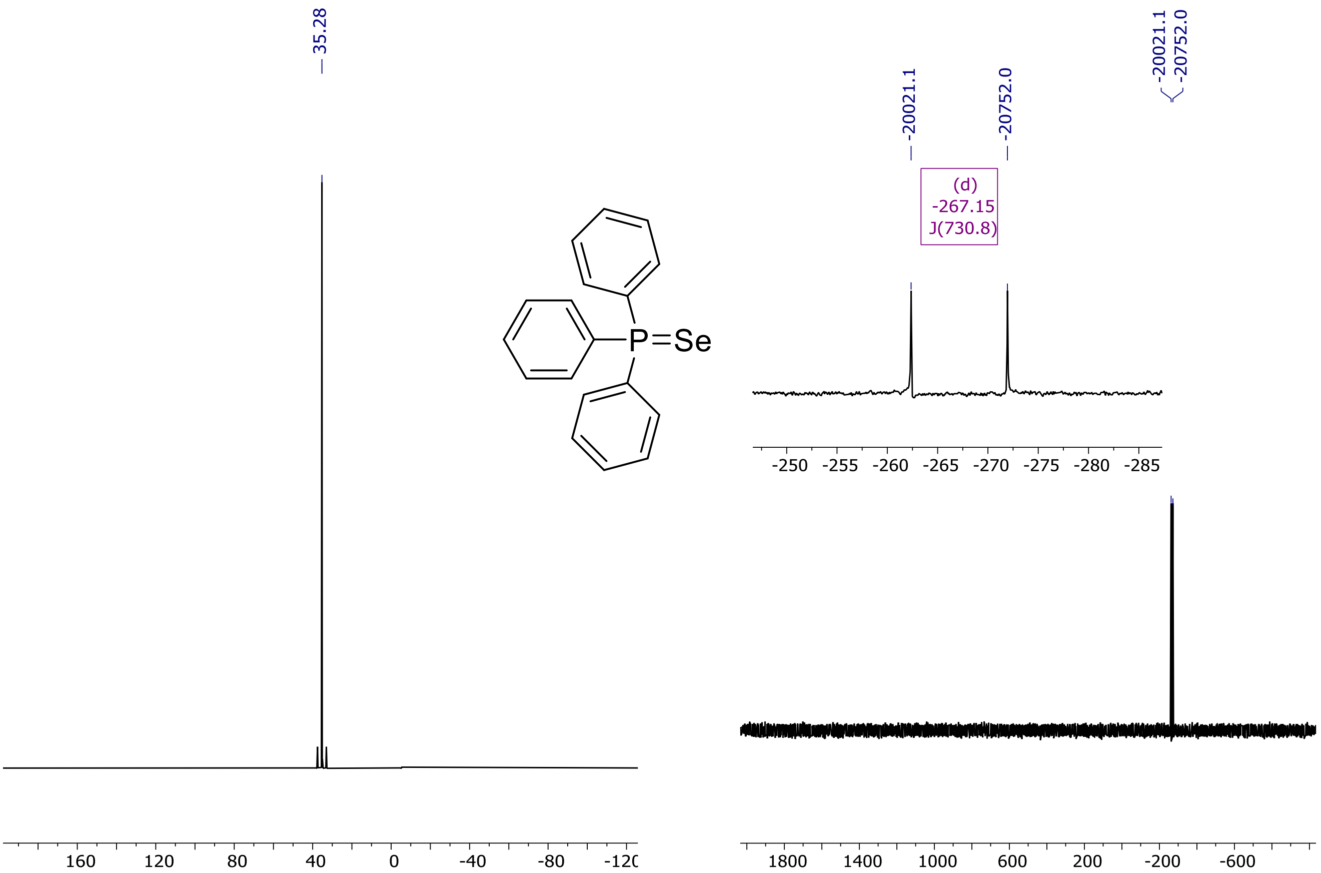

Figure S6. ^{31}P NMR spectrum (left,162 MHz,) and ^{77}Se NMR spectrum (right, 76 MHz) of triphenylphosphane selenide (RT, $CDCl_3$)

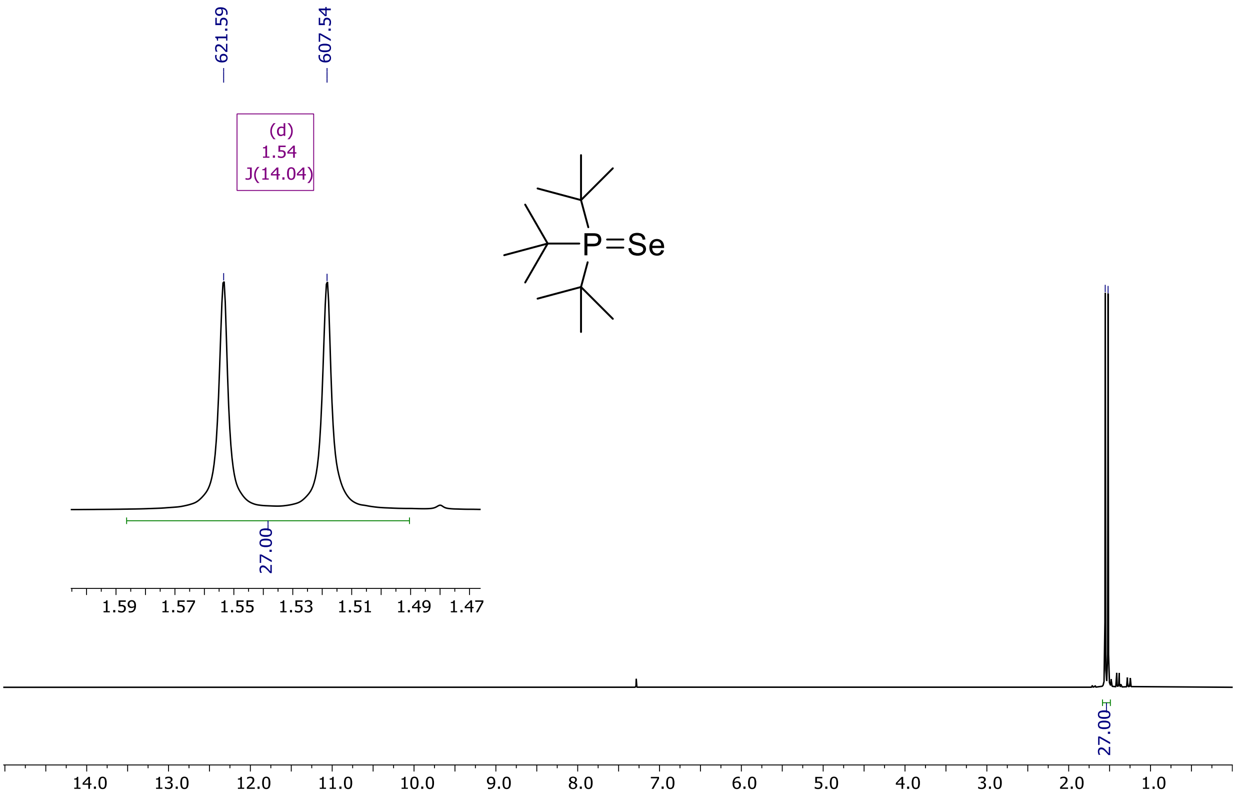

Figure S7. ^{1}H NMR spectrum of tris(*tert*-butyl)phosphane selenide (400 MHz, RT, $CDCl_3$)

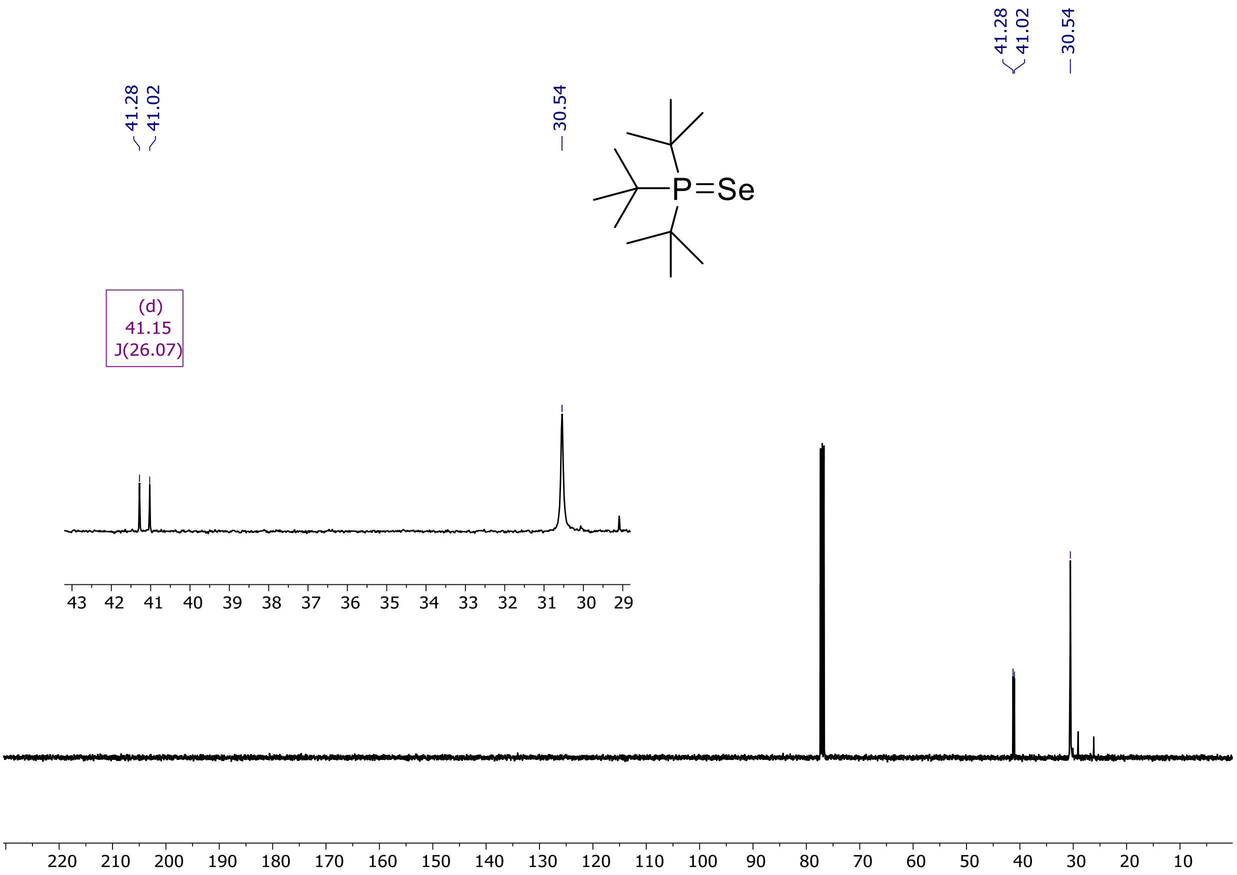

Figure S8. ^{13}C NMR spectrum of tris(*tert*-butyl)phosphane selenide (101 MHz, RT, $CDCl_3$)

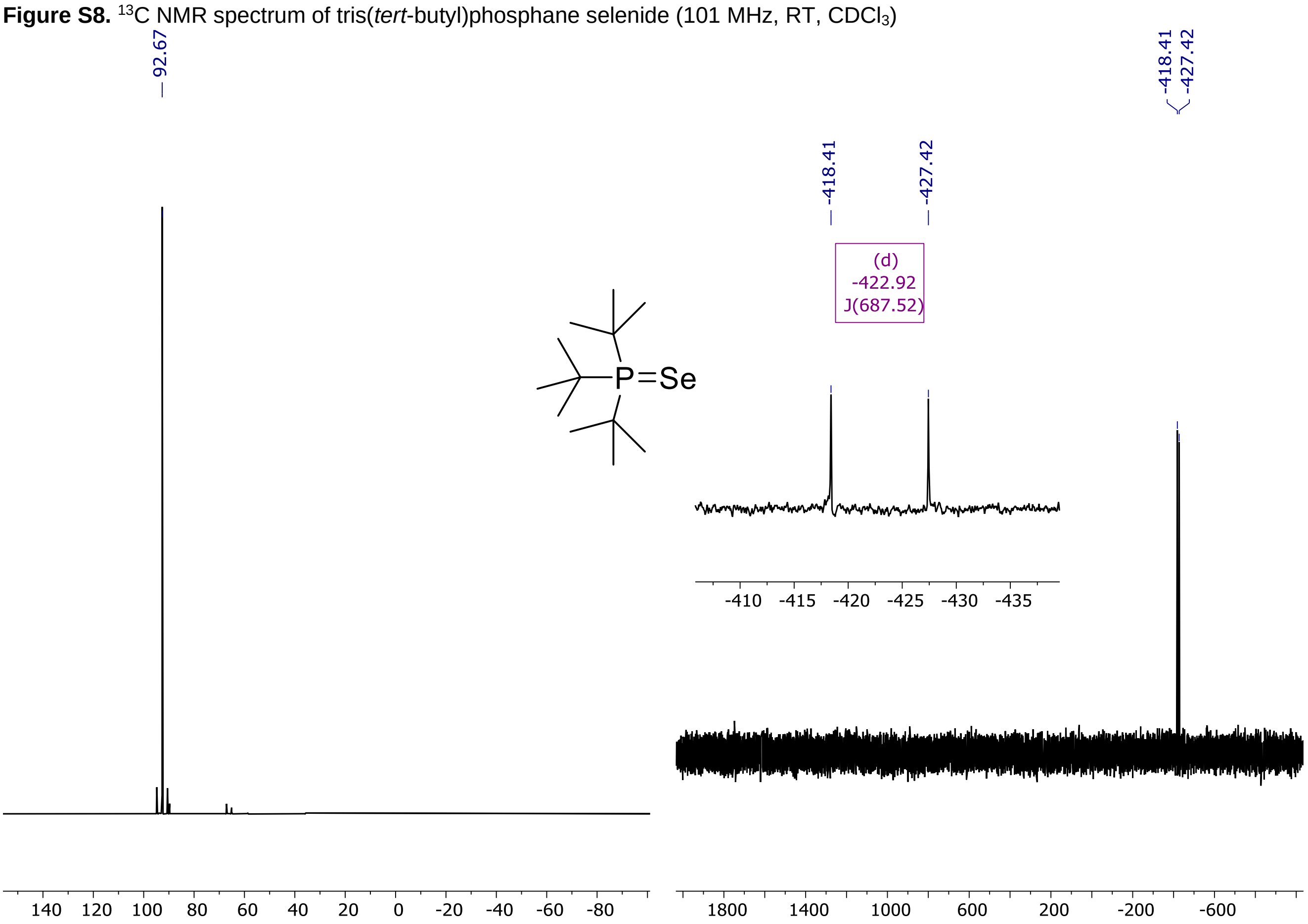

Figure S9. ^{31}P NMR spectrum (left,162 MHz,) and ^{77}Se NMR spectrum (right, 76 MHz) of tris(*tert*-butyl)phosphane selenide (RT, $CDCl_3$)

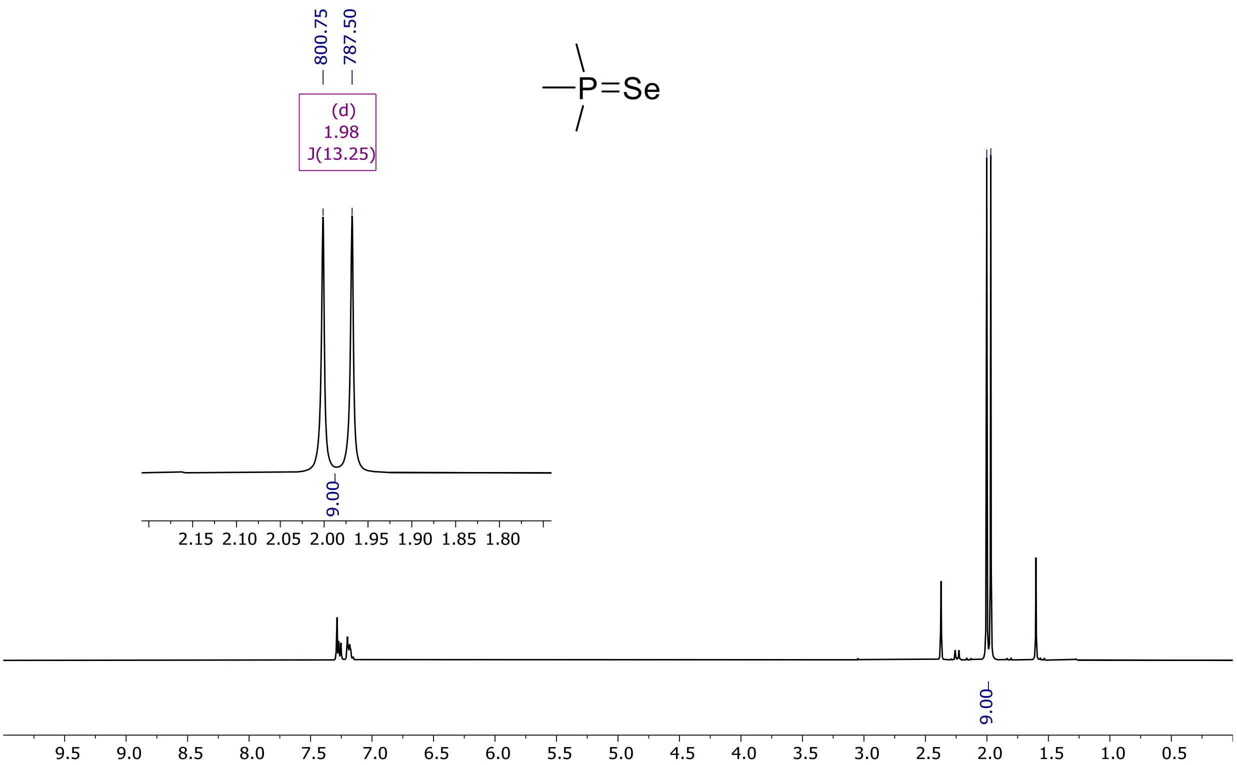

Figure S10. ^{1}H NMR spectrum of trimethylphosphane selenide (400 MHz, RT, $CDCl_3$)

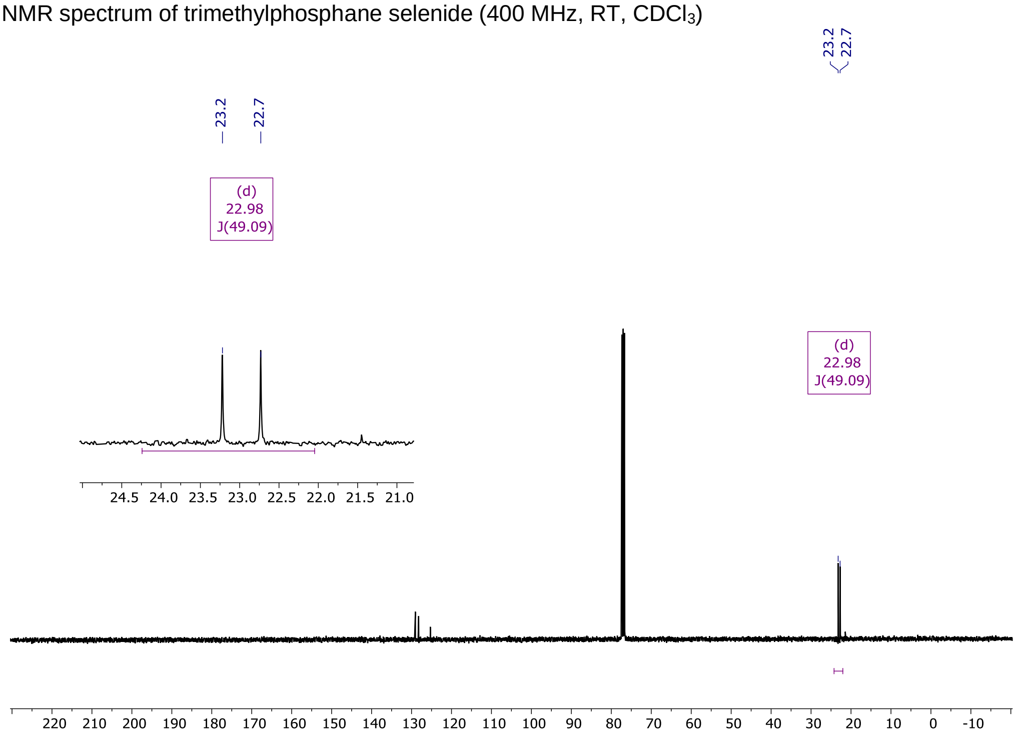

Figure S11. ^{13}C NMR spectrum of trimethylphosphane selenide (101 MHz, RT, $CDCl_3$)

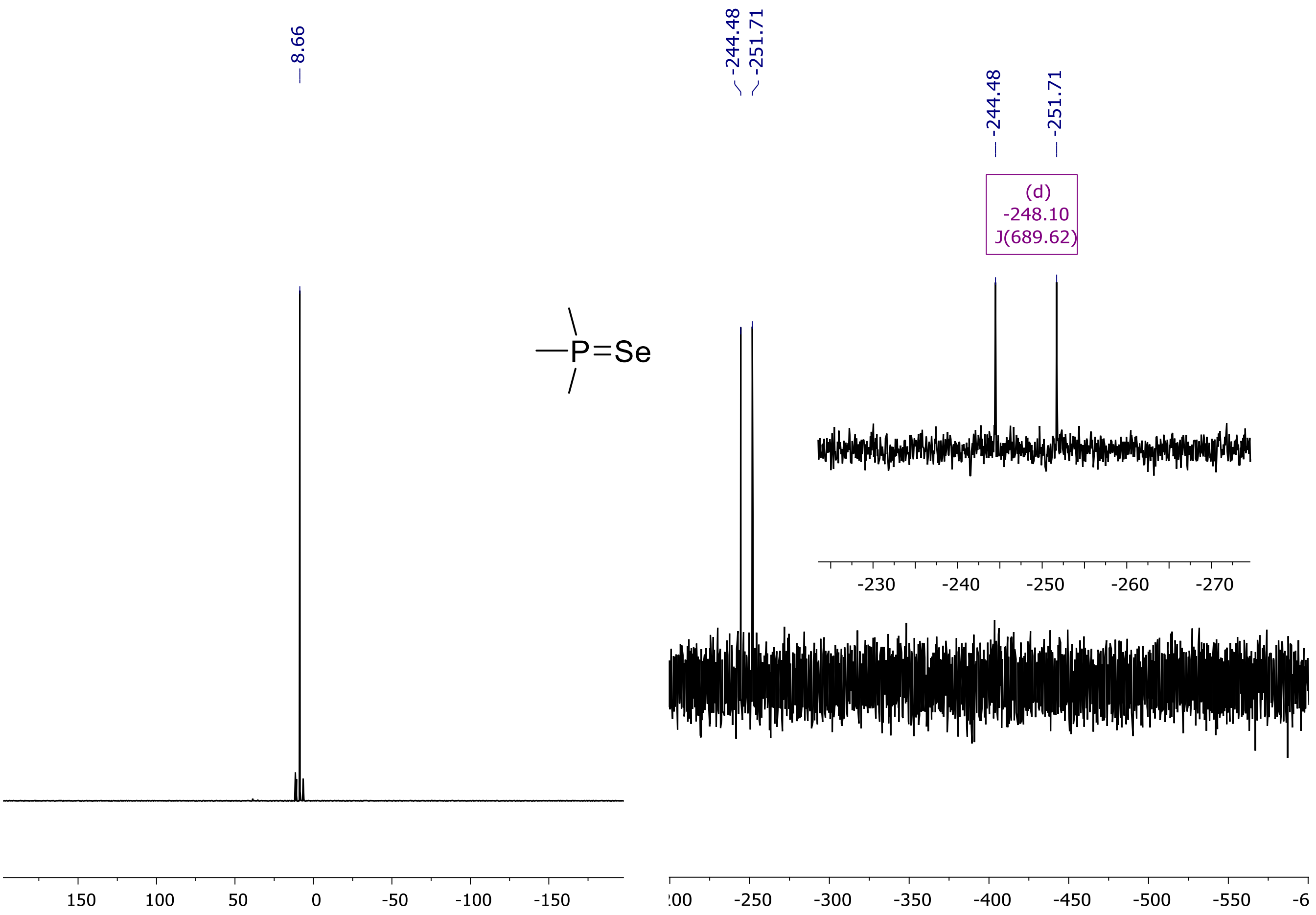

Figure S12. ^{31}P NMR spectrum (left,162 MHz,) and ^{77}Se NMR spectrum (right, 76 MHz) of trimethylphosphane selenide (RT, $CDCl_3$)

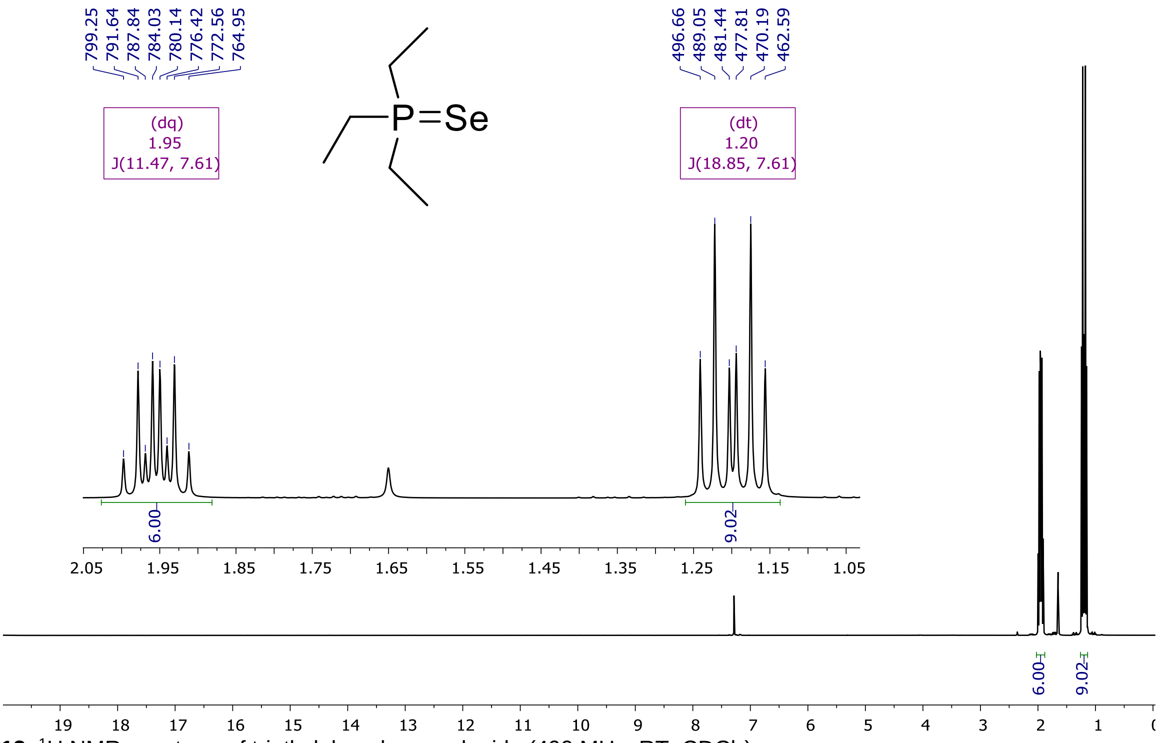

Figure S13. ^{1}H NMR spectrum of triethylphosphane selenide (400 MHz, RT, $CDCl_3$)

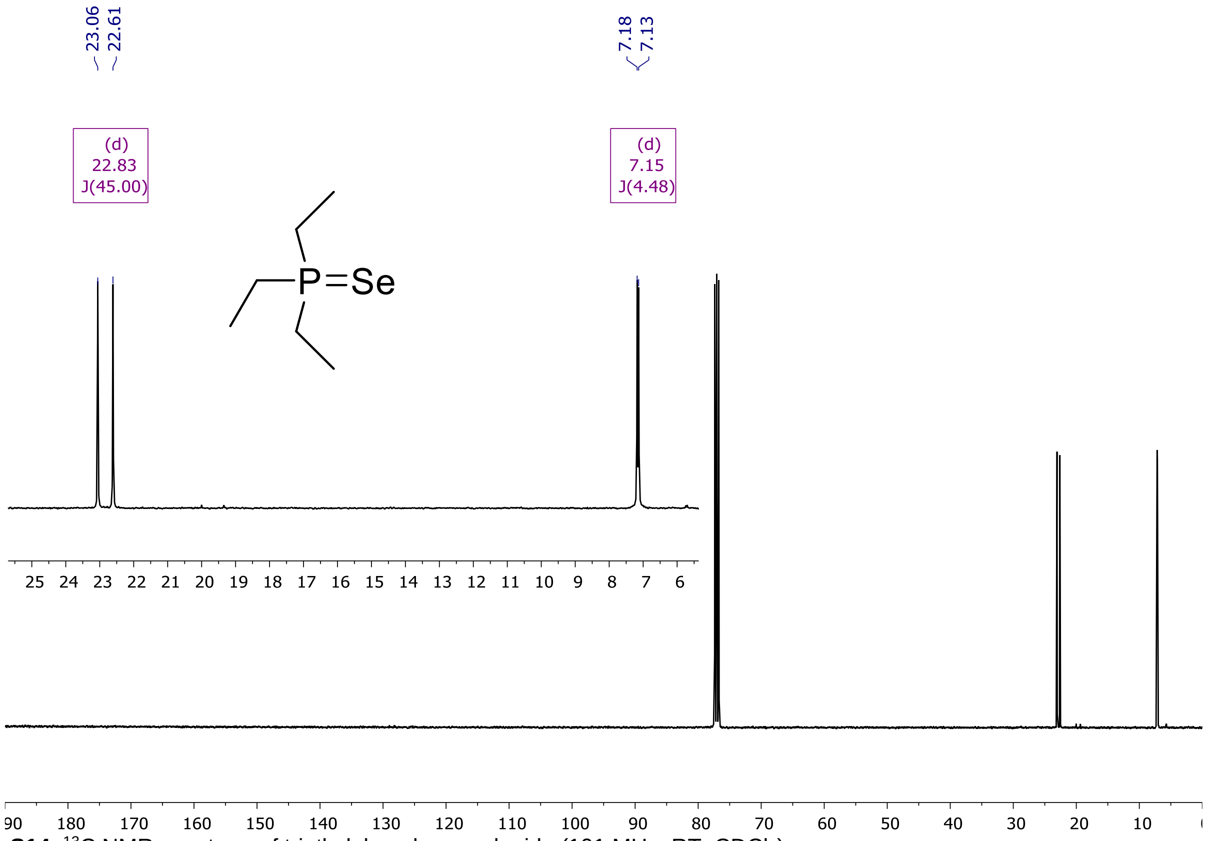

Figure S14. ^{13}C NMR spectrum of triethylphosphane selenide (101 MHz, RT, $CDCl_3$)

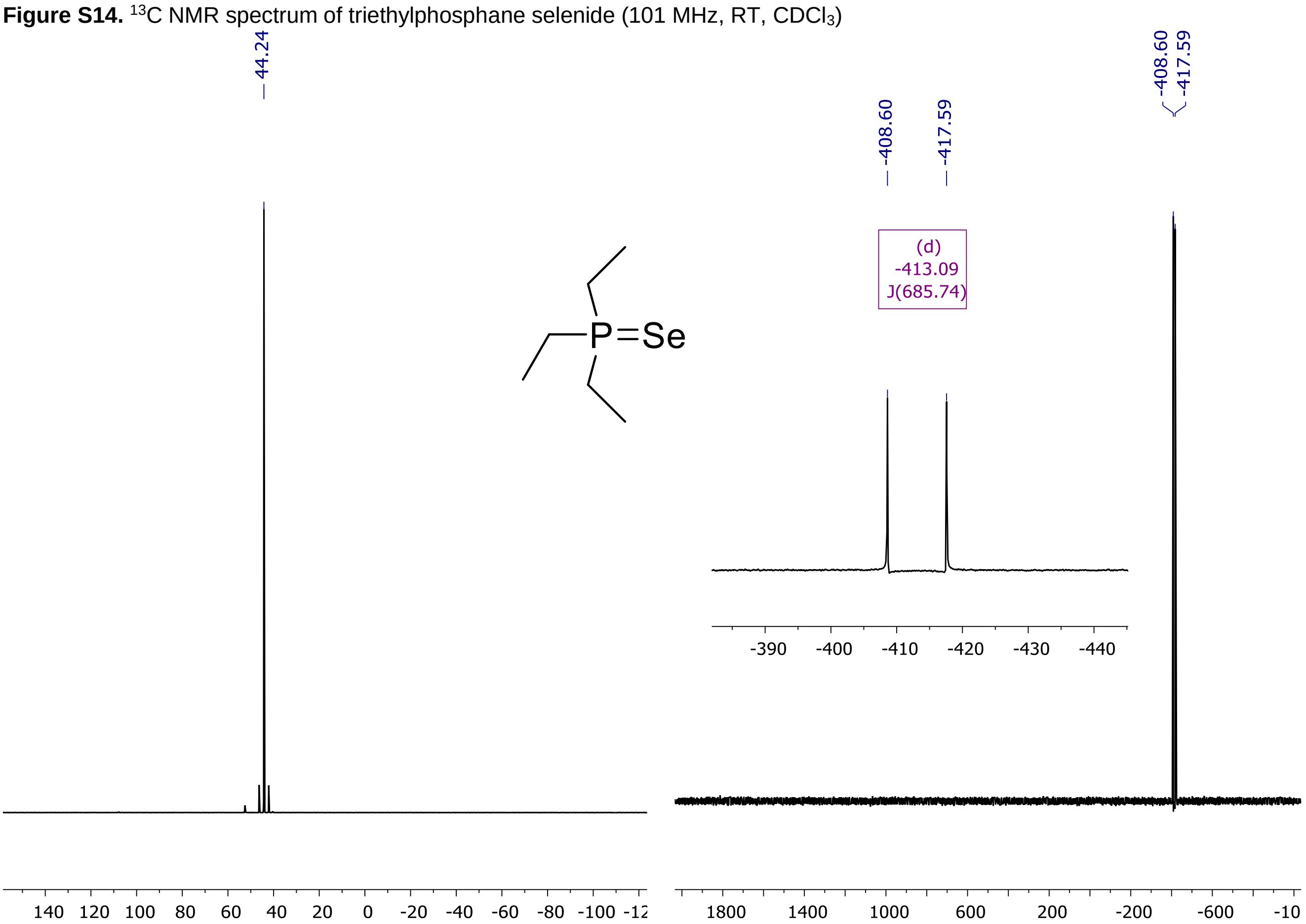

Figure S15. ^{31}P NMR spectrum (left,162 MHz,) and ^{77}Se NMR spectrum (right, 76 MHz) of triethylphosphane selenide (RT, $CDCl_3$)

Copies of ^{1}H and ^{13}C NMR spectra of arsine oxides R_3AsO

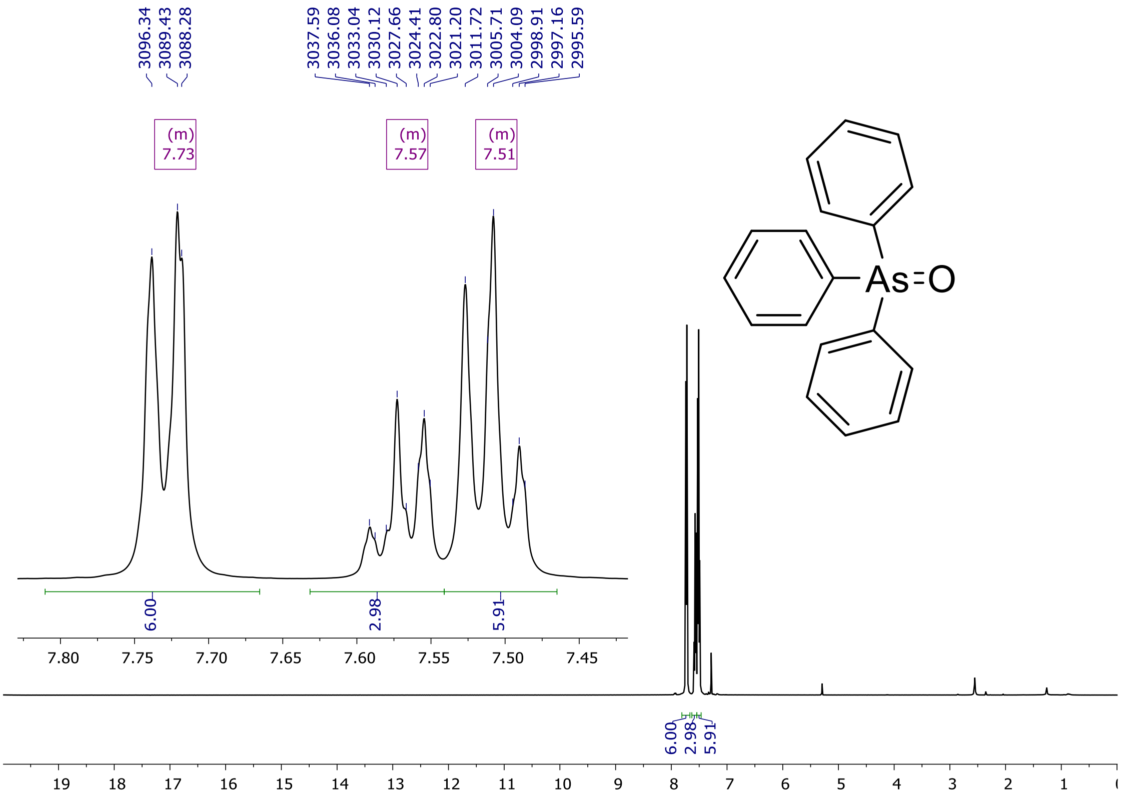

Figure S16. ^{1}H NMR spectrum of triphenylarsane oxide (400 MHz, RT, $CDCl_3$)

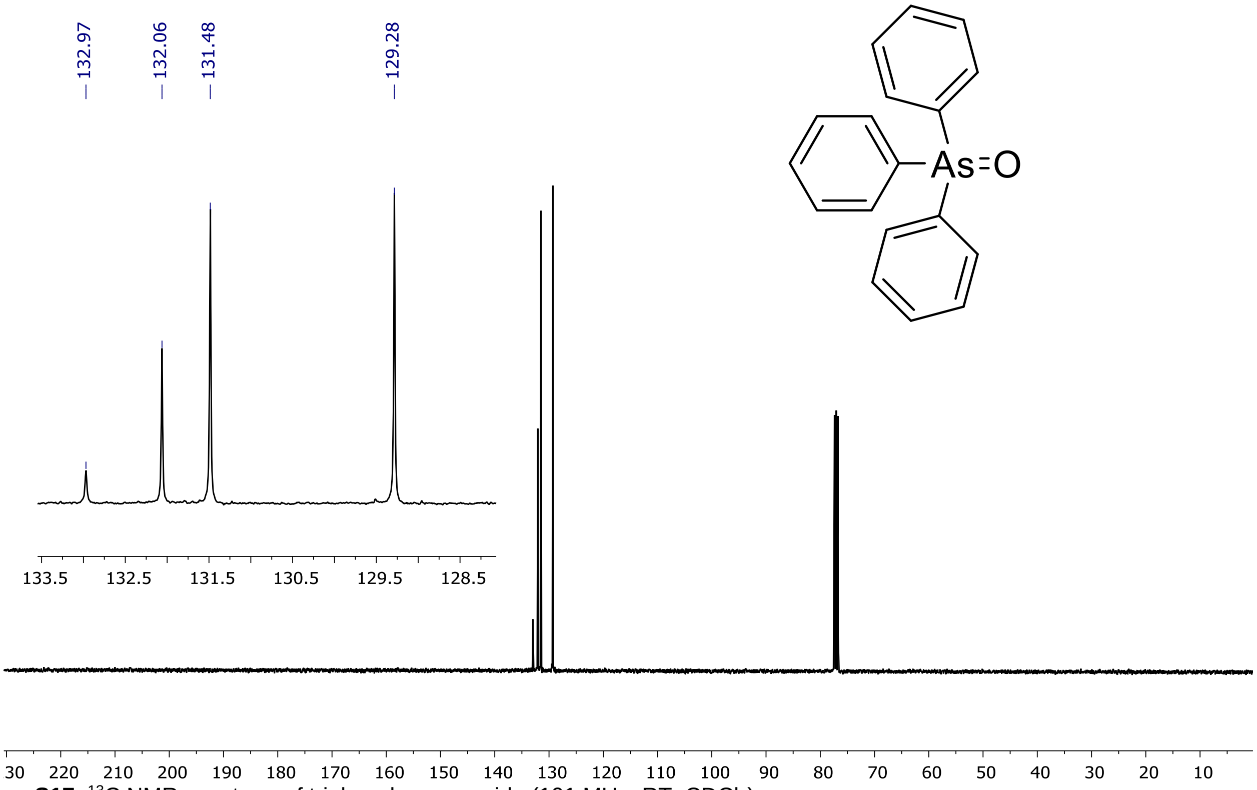

Figure S17. ^{13}C NMR spectrum of triphenylarsane oxide (101 MHz, RT, $CDCl_3$)

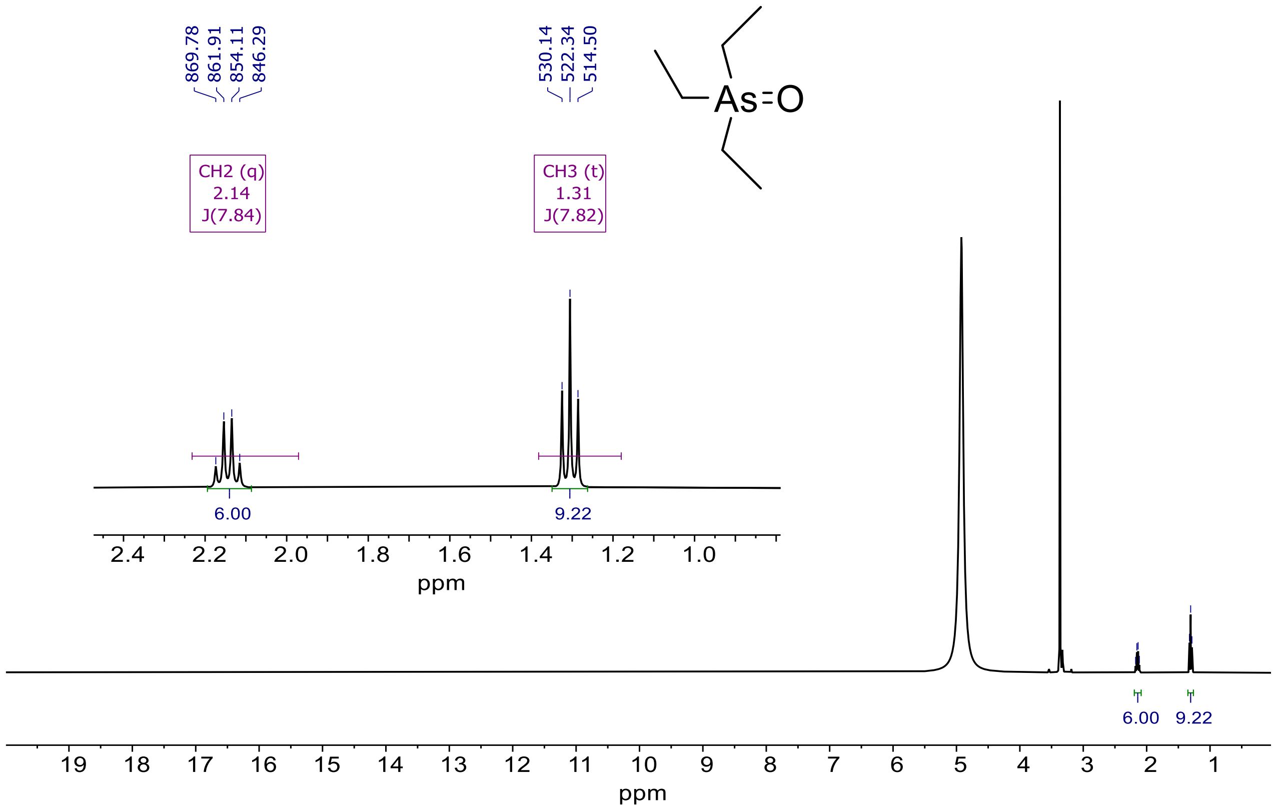

Figure S18. ^{1}H NMR spectrum of triethylarsane oxide (400 MHz, RT, CD_3OH)

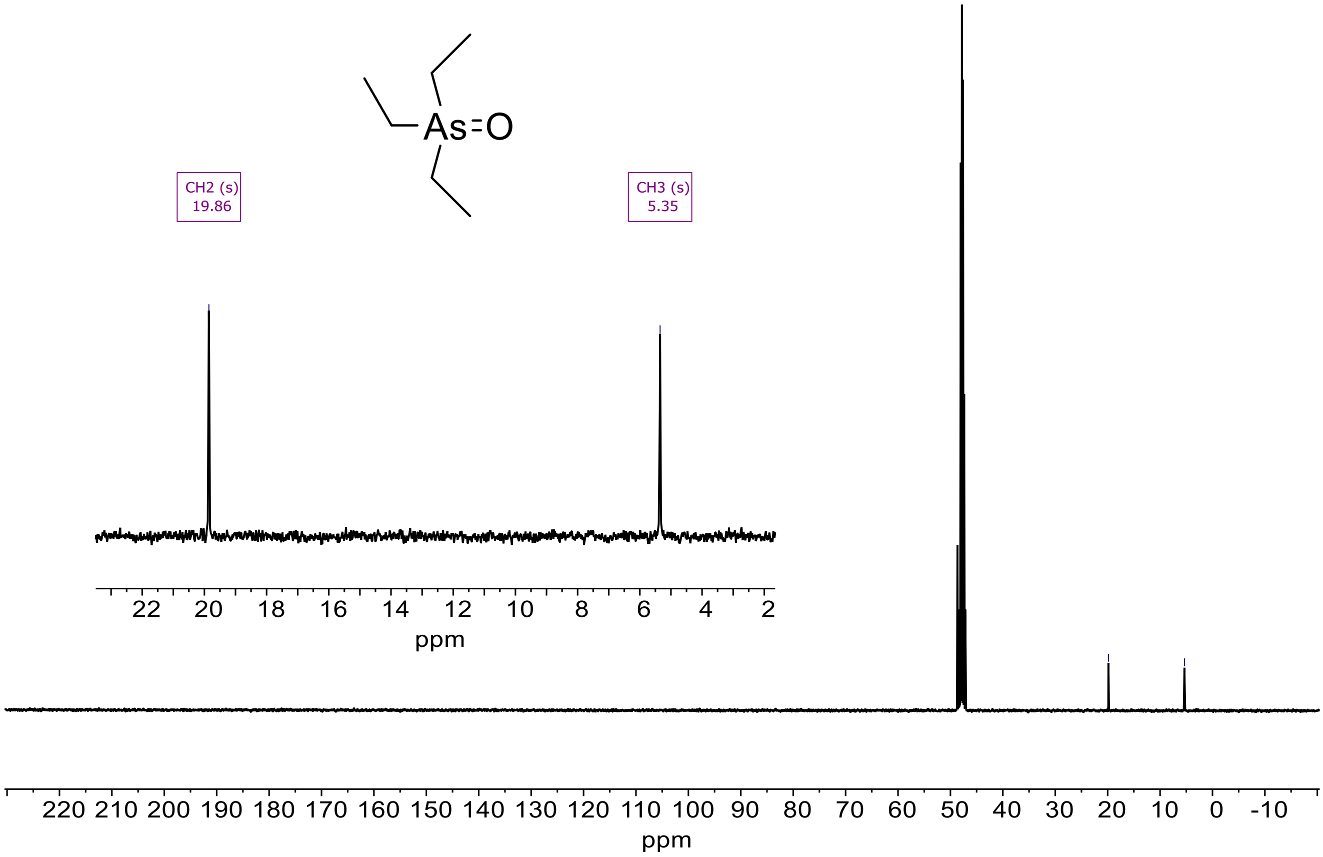

Figure S19. ^{13}C NMR spectrum of triethylarsane oxide (101 MHz, RT, CD_3OH)

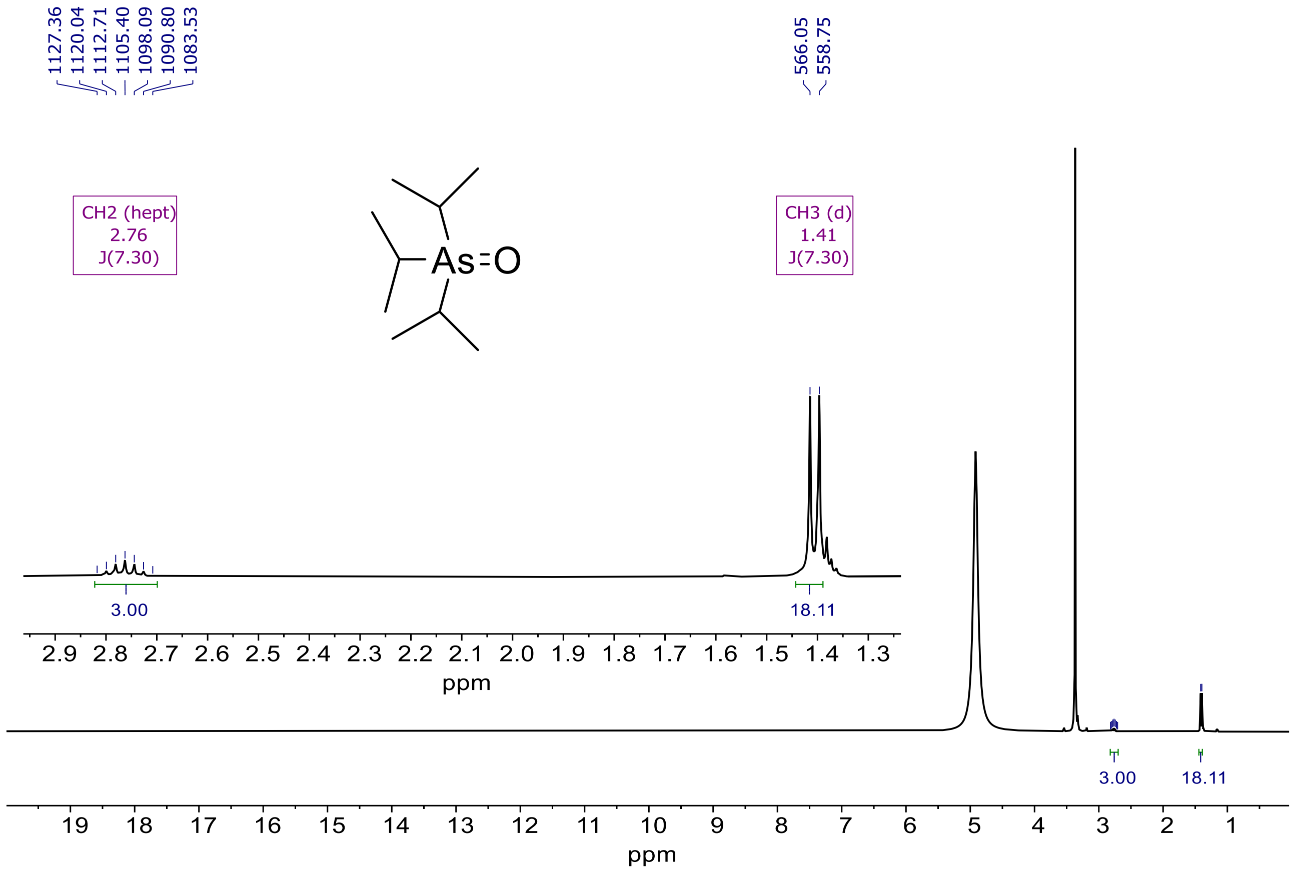

Figure S20. ^{1}H NMR spectrum of tri(*iso*-propyl)arsane oxide (400 MHz, RT, CD_3OH)

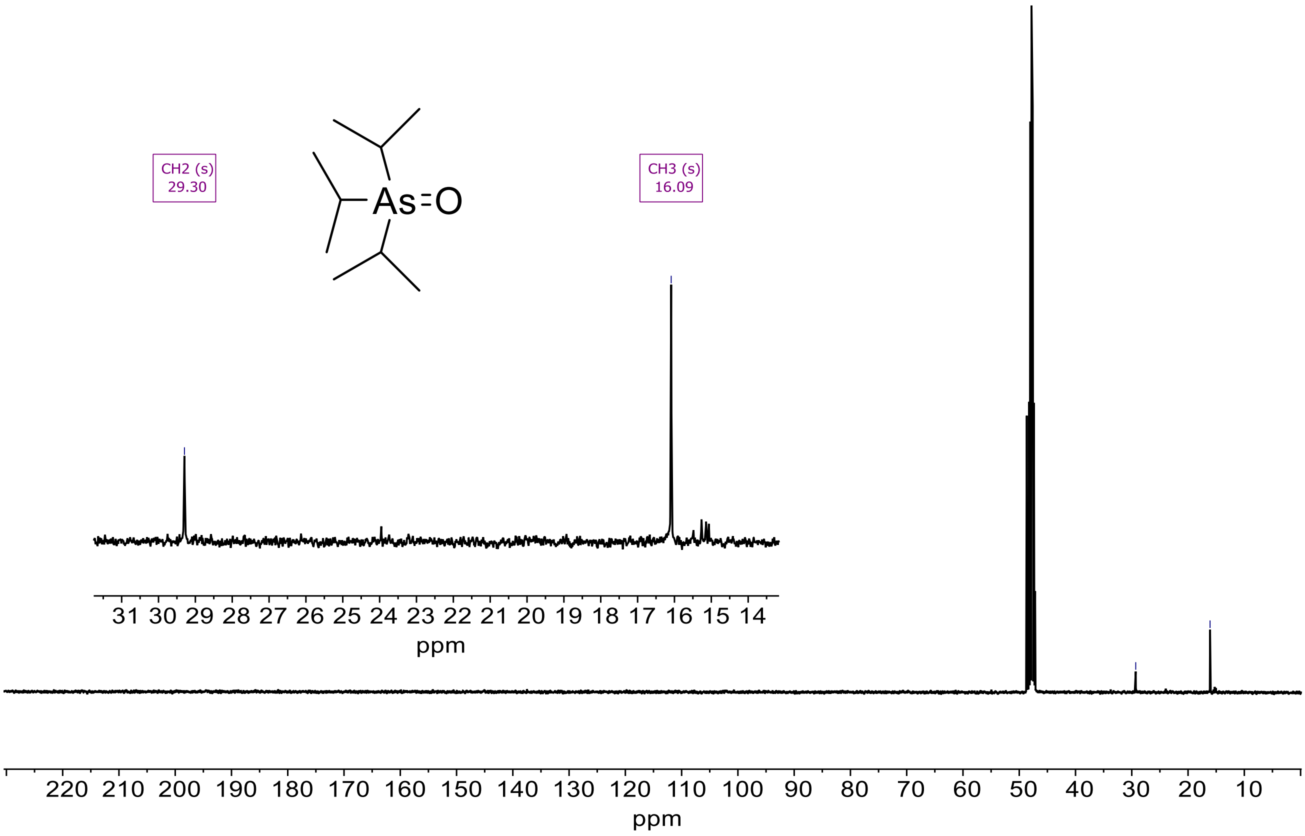

Figure S21. ^{13}C NMR spectra of tri(*iso*-propyl)arsane oxide (101 MHz, RT, CD_3OH)

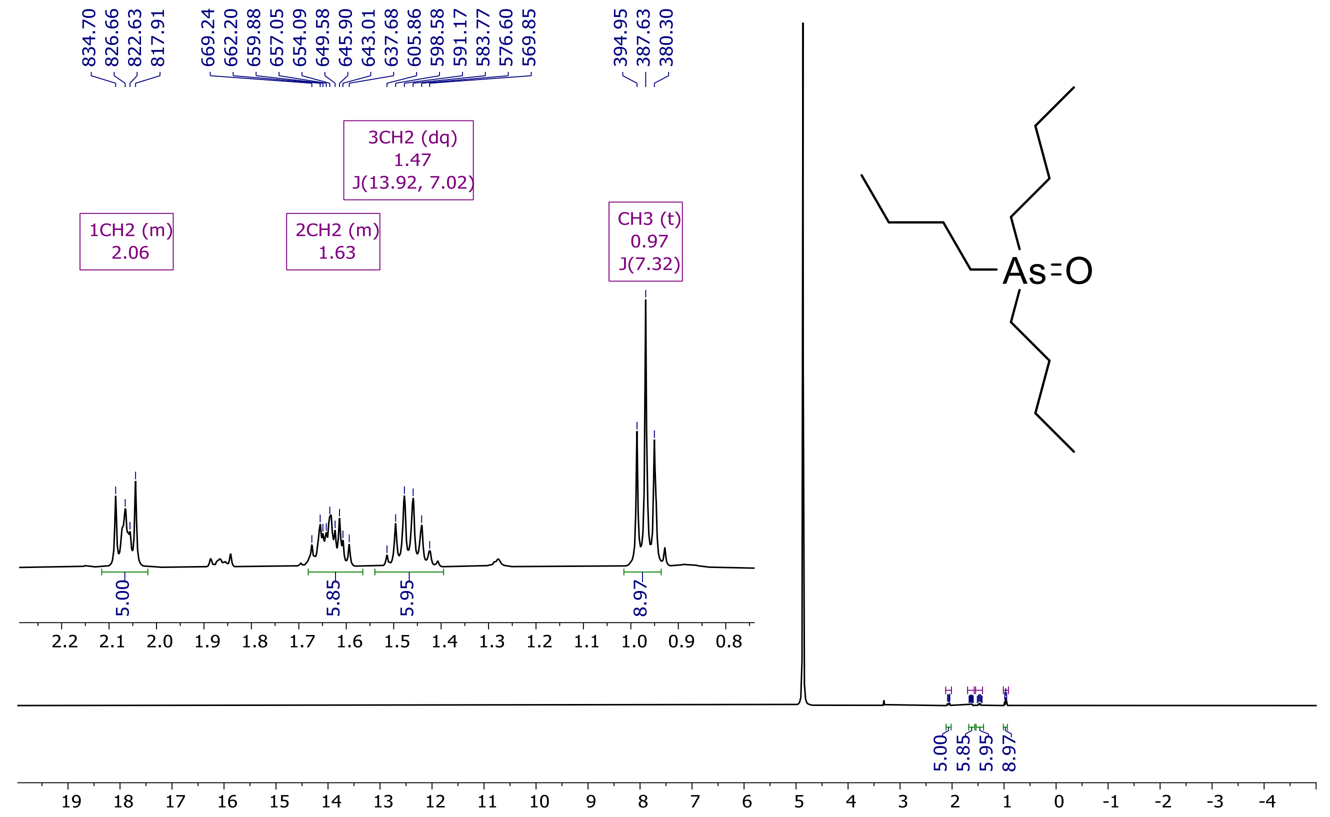

Figure S22. ^{1}H NMR spectrum of tri(*n*-butyl)arsane oxide (400 MHz, RT, CD_3OH)

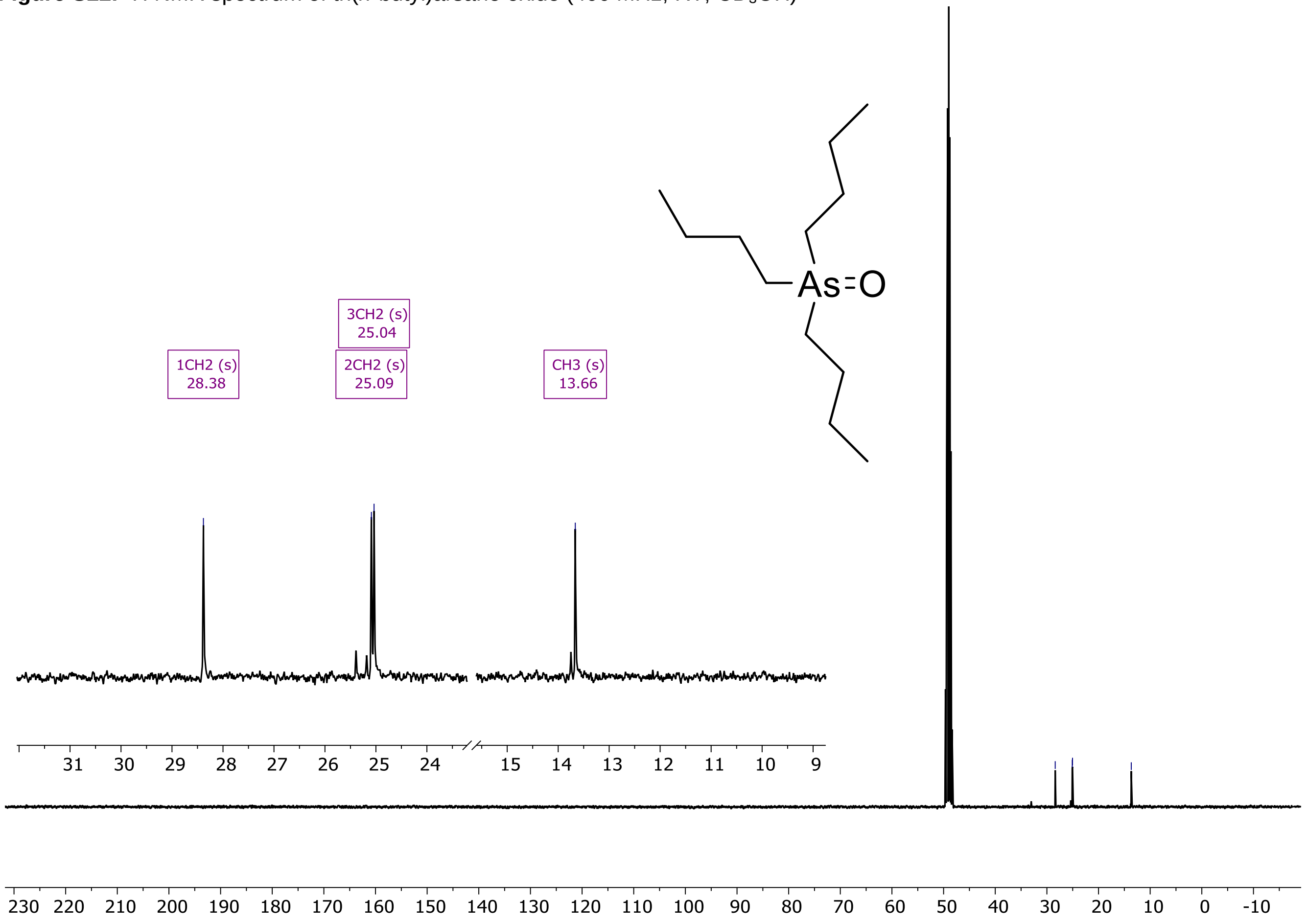

Figure S23. ^{13}C NMR spectra of tri(*n*-butyl)arsane oxide (101 MHz, RT, CD_3OH)

Copies of ^{1}H, ^{13}C and ^{77}Se NMR spectra of arsine selenides R_3AsSe

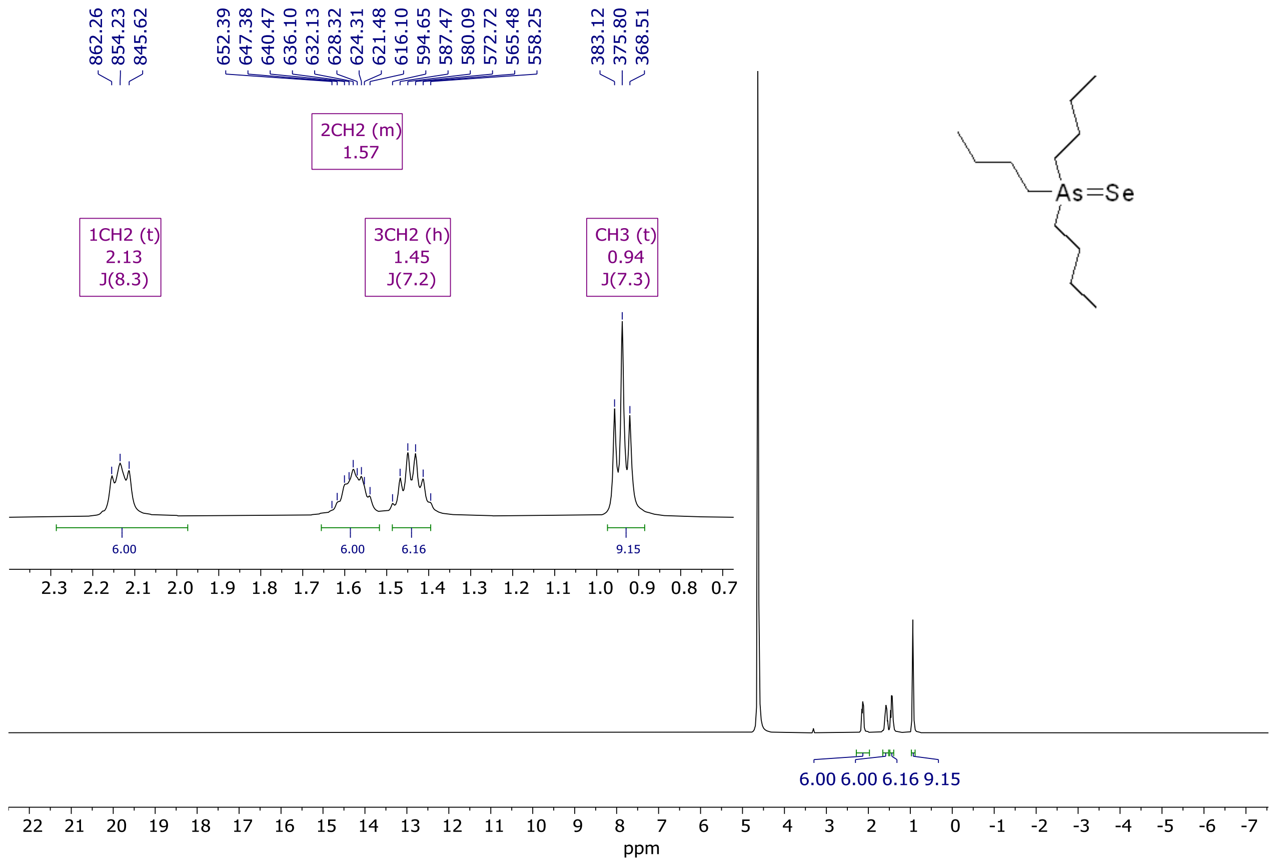

Figure S24. ^{1}H NMR spectrum of tri(*n*-butyl)arsane selenide (400 MHz, RT, CD_3OH)

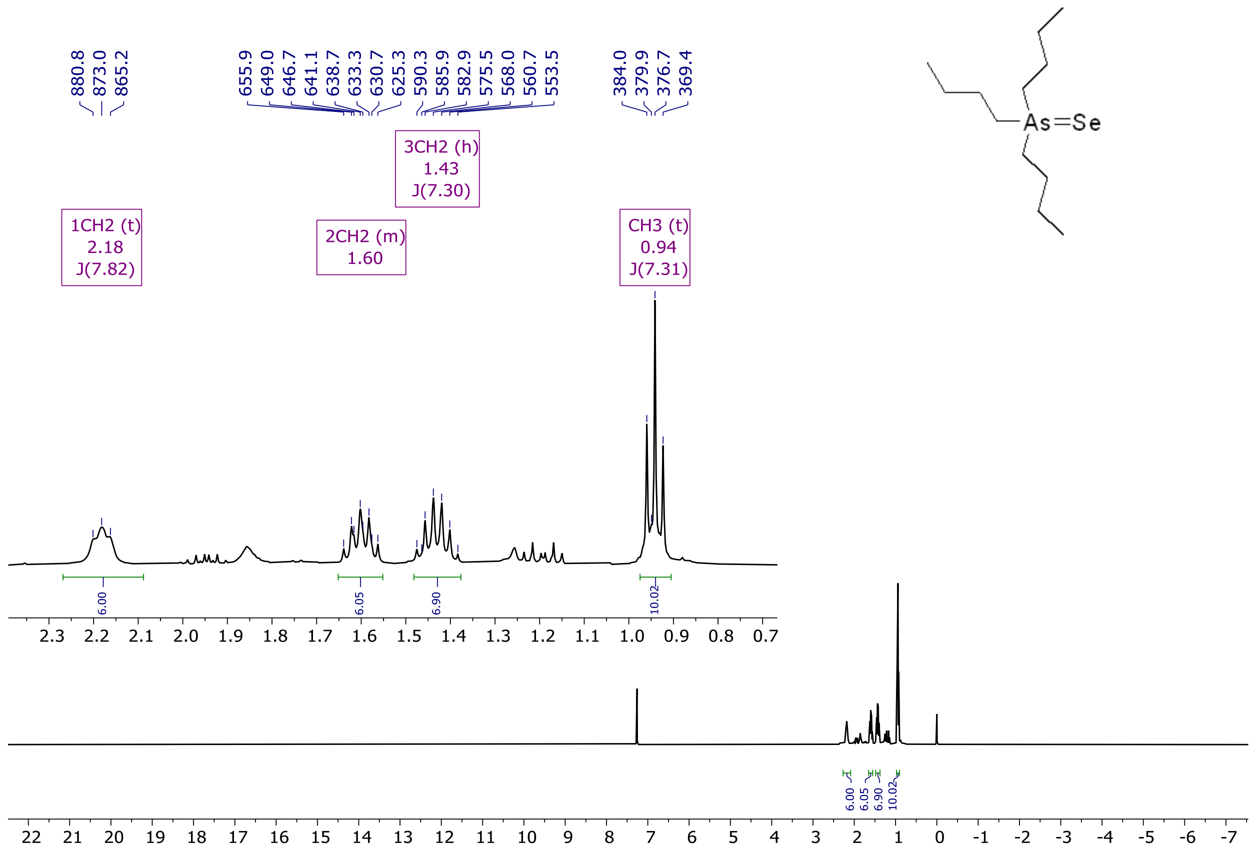

Figure S25. ^{1}H NMR spectrum of tri(*n*-butyl)arsane selenide (400 MHz, RT, $CDCl_3$)

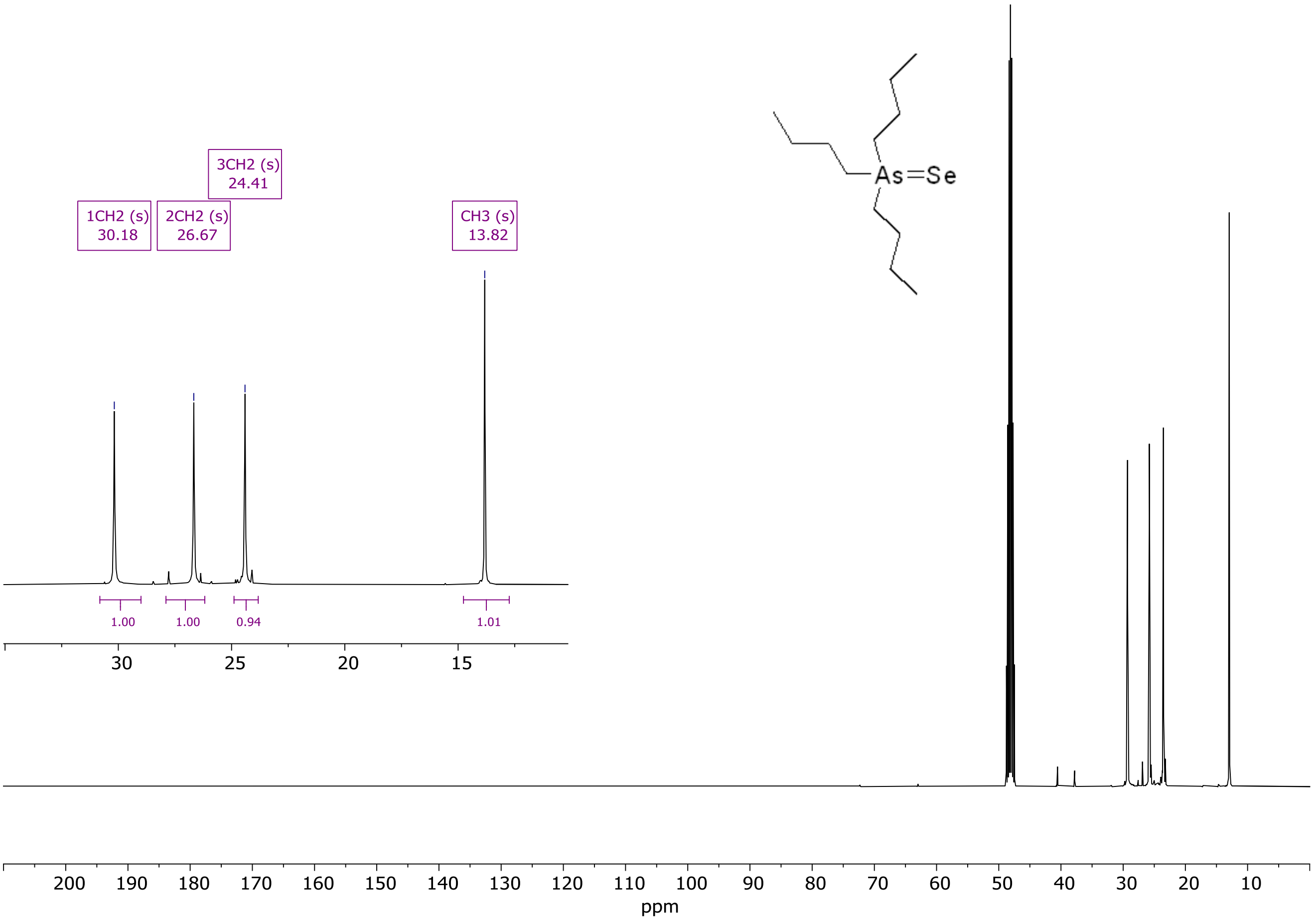

Figure S26. ^{13}C NMR spectra of tri(*n*-butyl)arsane selenide (101 MHz, RT, CD_3OH)

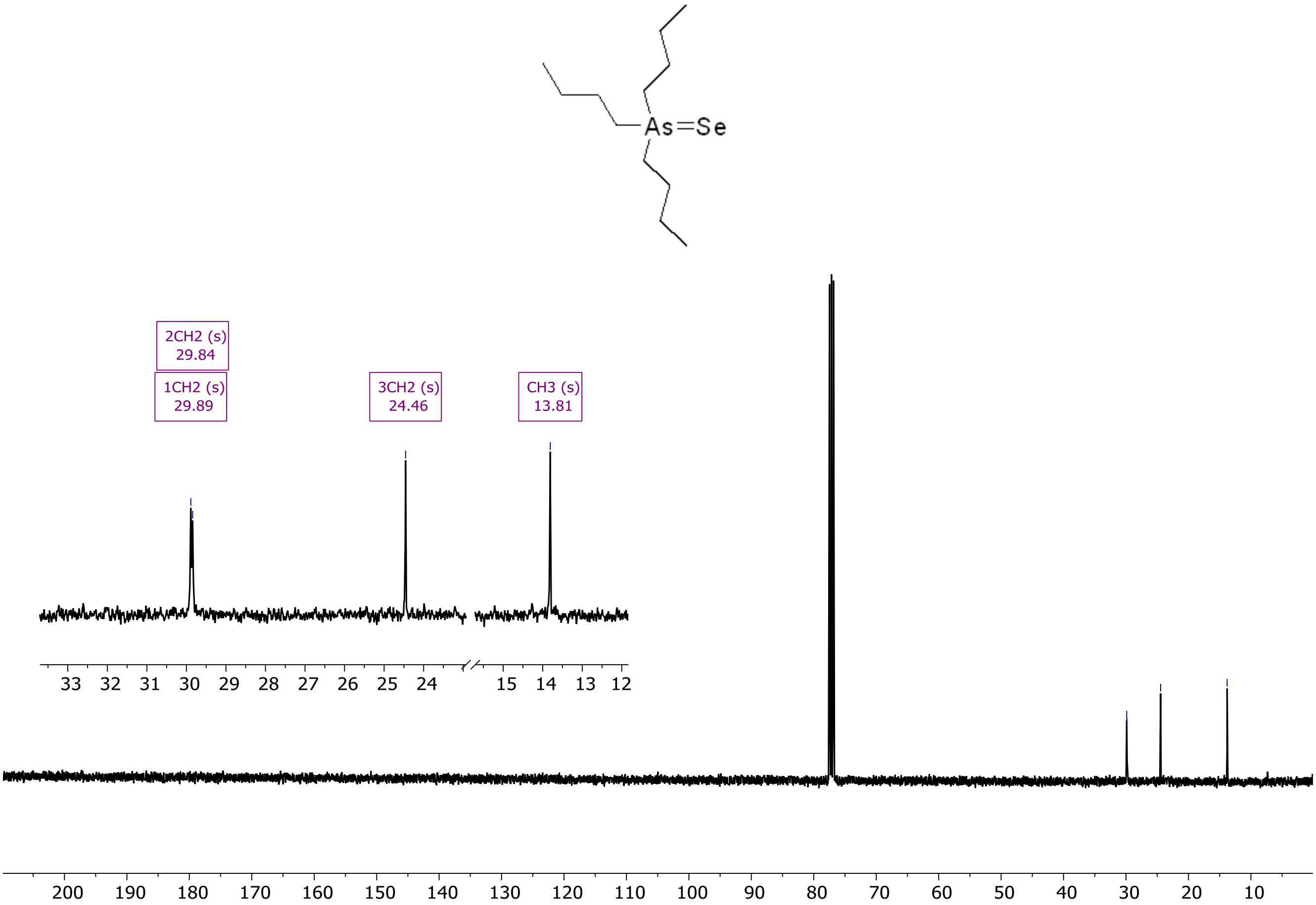

Figure S27. ^{13}C NMR spectra of tri(*n*-butyl)arsane selenide (101 MHz, RT, $CDCl_3$)

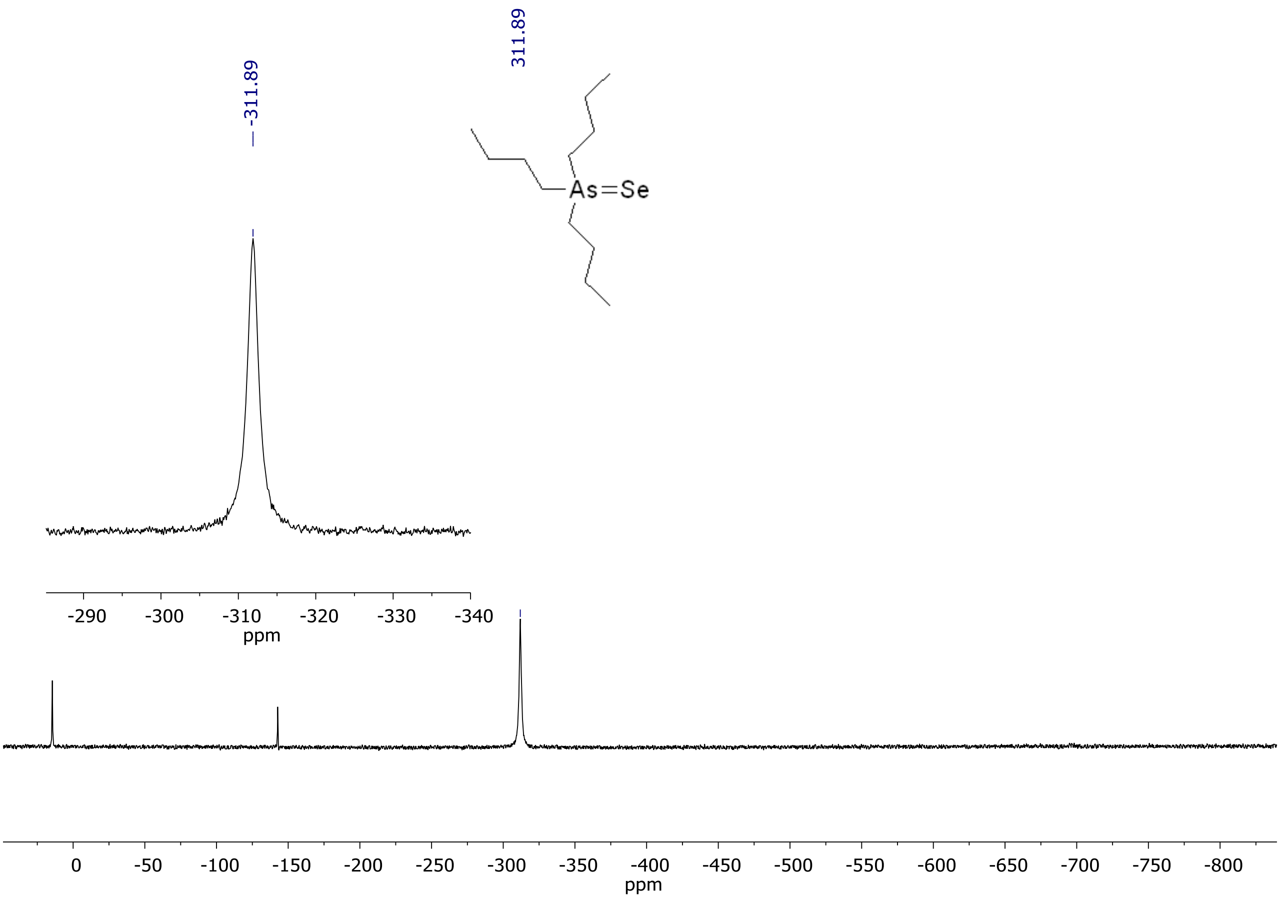

Figure S28. ^{77}Se NMR spectrum of tri(*n*-butyl)arsane selenide (76 MHz, RT, CD_3OH)

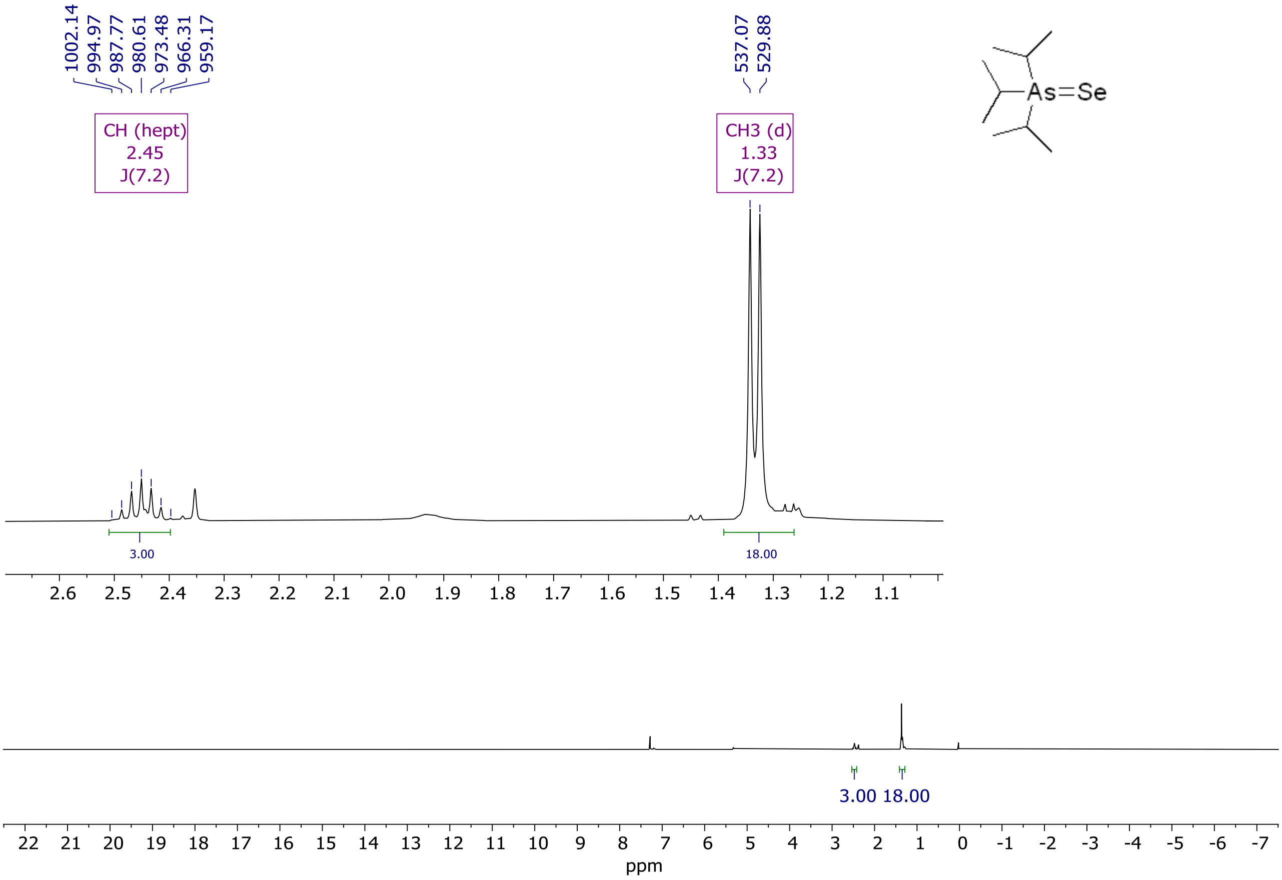

Figure S29. ^{1}H NMR spectrum of tri(*iso*-propyl)arsane selenide (400 MHz, RT, $CDCl_3$)

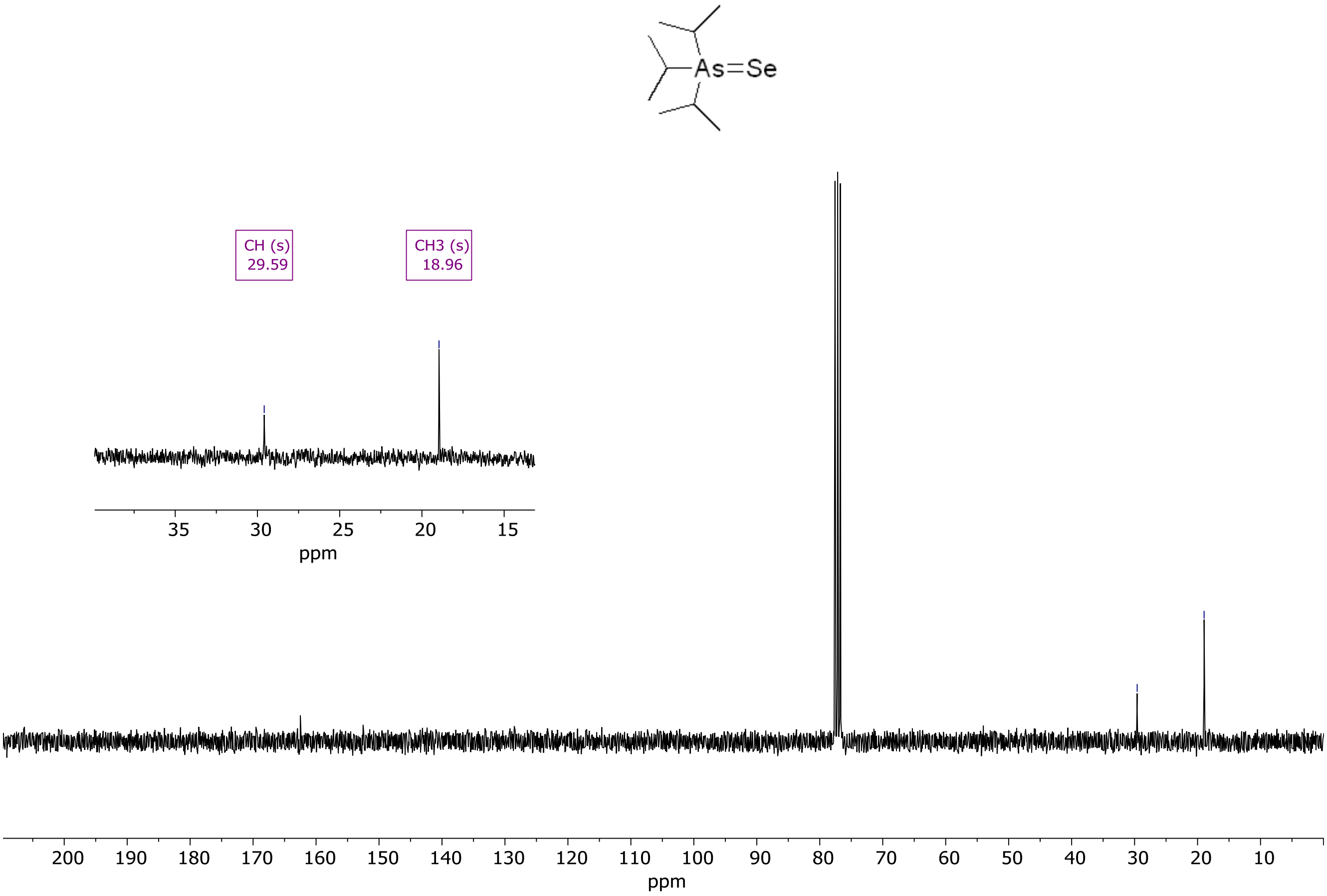

Figure S30. ^{13}C NMR spectra of tri(*iso*-propyl)arsane selenide (101 MHz, RT, $CDCl_3$)

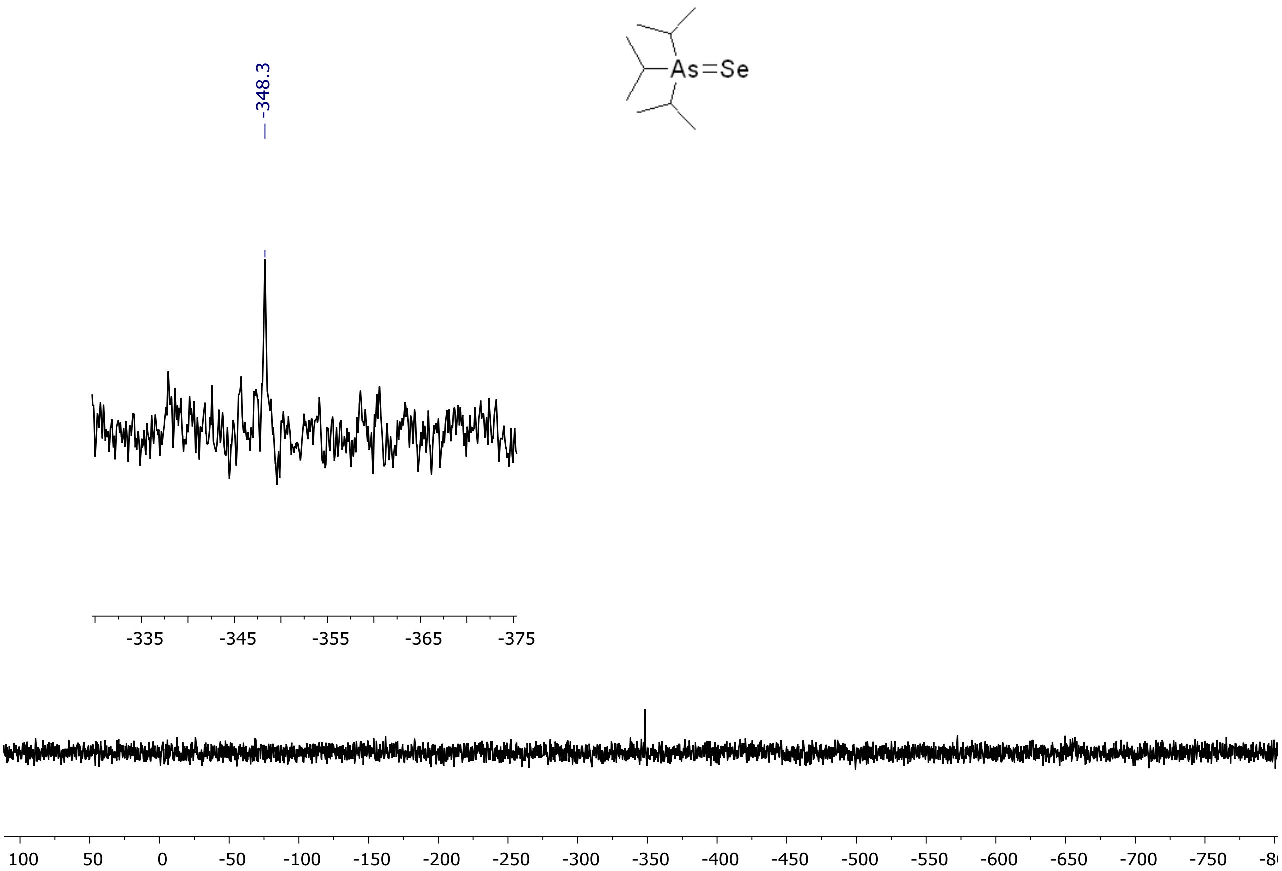

Figure S31. ^{77}Se NMR spectrum of tri(*iso*-propyl)arsane selenide (76 MHz, RT, CD_3OH)

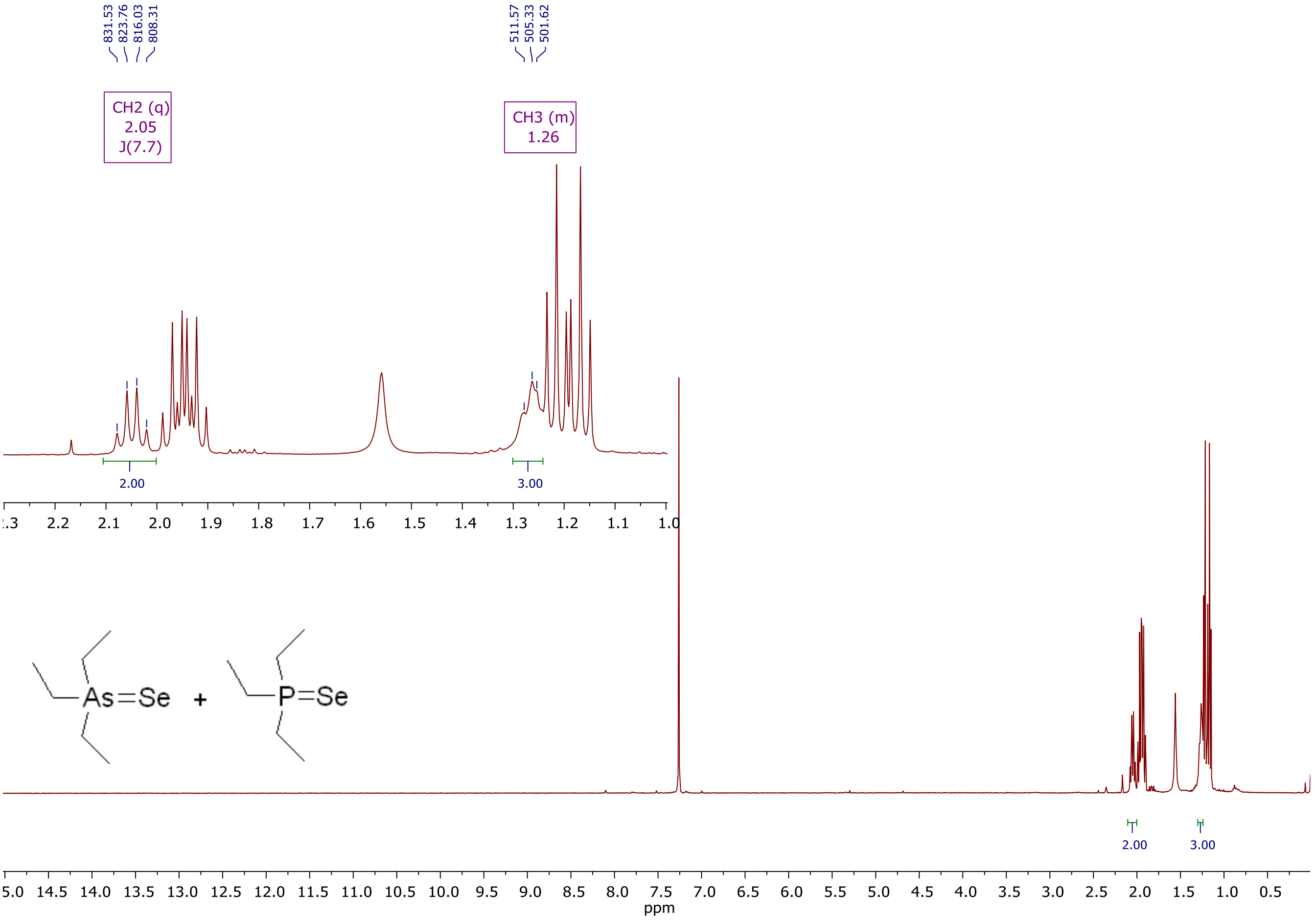

Figure S32. ^{1}H NMR spectra of triethylarsane selenide with unseparatable admixture of trhiethylphosphane selenide (400 MHz, RT, $CDCl_3$)

X-ray studies

Table S3. Crystal data and structure refinement for.

Identification code	**Ph_3AsO···Phenol**	**Ph_3AsO···4-F-Phenol**
CCDC code	2440095	2440099
Empirical formula	C54H48As2O5	C27H22.45AsF1.54O2.46
Formula weight	927.34	490.46
Temperature, K	100(2)	100(2)
Crystal system	triclinic	triclinic
Space group	P-1	P-1
a, Å	8.41510(10)	8.5914(3)
b, Å	9.67240(10)	9.8180(4)
c, Å	14.9436(2)	14.7479(6)
α, °	104.6010(10)	105.057(4)
β, °	100.3710(10)	101.956(3)
γ, °	102.0280(10)	100.482(3)
Volume, $Å^3$	1115.91(2)	1138.04(8)
Z	1	2
ρ_{calc}, g/cm^3	1.380	1.431
μ, mm^{-1}	2.240	2.328
F(000)	478.0	502.0
Crystal size, mm^3	0.13 × 0.1 × 0.05	0.12 × 0.08 × 0.06
Radiation	CuKα (λ = 1.54184)	CuKα (λ = 1.54184)
2Θ range for data collection, °	6.306 to 160.186	6.442 to 152.596
Index ranges	-10 ≤ h ≤ 10, -12 ≤ k ≤ 12, -18 ≤ l ≤ 19	-7 ≤ h ≤ 10, -12 ≤ k ≤ 12, -17 ≤ l ≤ 18
Reflections collected	14527	8996
Independent reflections	4679 [R_{int} = 0.0324, R_{sigma} = 0.0328]	4653 [R_{int} = 0.0504, R_{sigma} = 0.0546]
Data/restraints/ parameters	4679/1/286	4653/6/292
Goodness-of-fit on F^2	1.083	1.033
Final R indexes [I≥2σ (I)]	R_1 = 0.0276, wR_2 = 0.0687	R_1 = 0.0376, wR_2 = 0.0958
Final R indexes [all data]	R_1 = 0.0291, wR_2 = 0.0698	R_1 = 0.0421, wR_2 = 0.0996
Largest diff. peak/ hole, e·$Å^{-3}$	0.34/-0.39	0.75/-0.65

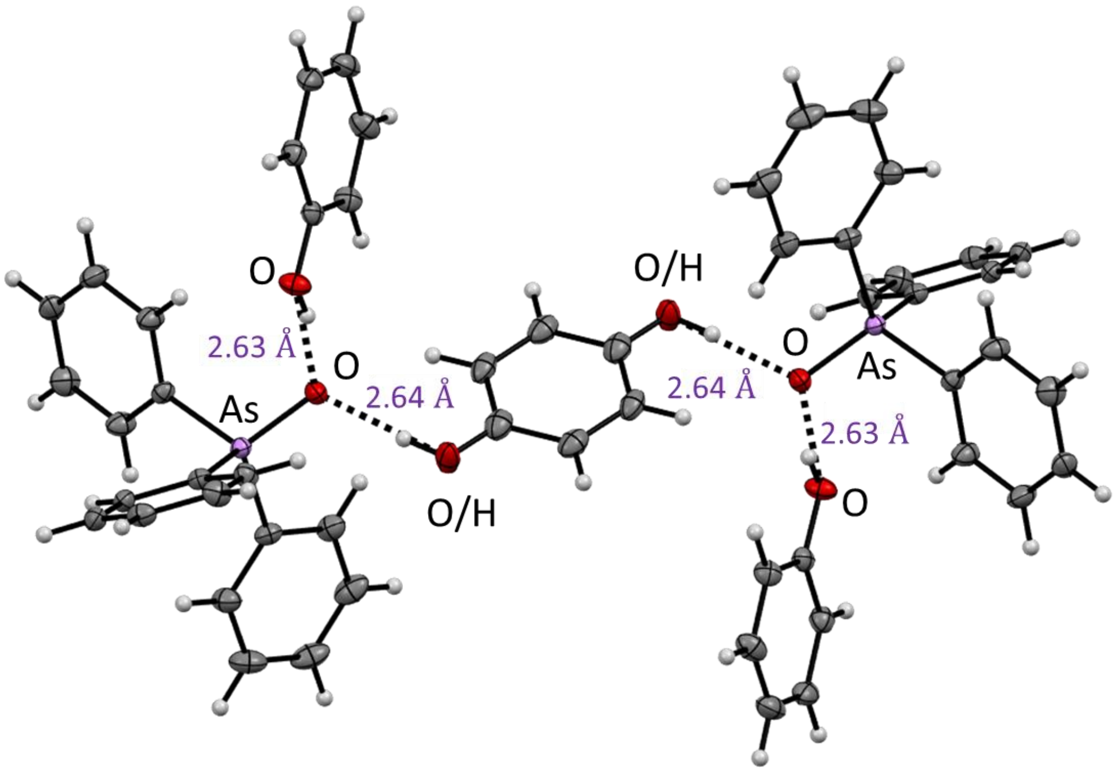

Figure S33. Molecular structure of the hydrogen bonded complex **$Ph_3AsO\cdots$Phenol.**

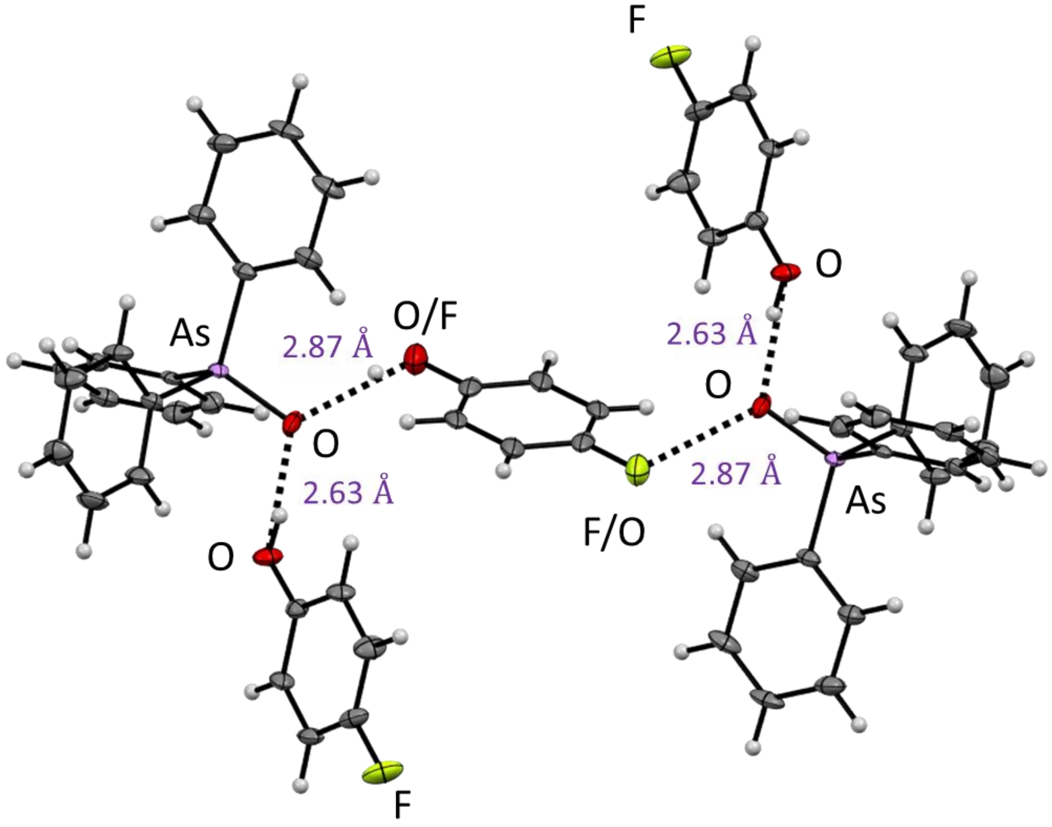

Figure S34. Molecular structure of the hydrogen bonded complex **$Ph_3AsO\cdots$4-F-Phenol**
